# A Temperature-Responsive Network Links Cell Shape and Virulence Traits in a Primary Fungal Pathogen

**DOI:** 10.1371/journal.pbio.1001614

**Published:** 2013-07-23

**Authors:** Sinem Beyhan, Matias Gutierrez, Mark Voorhies, Anita Sil

**Affiliations:** 1 Department of Microbiology and Immunology, University of California, San Francisco, San Francisco, California, United States of America; 2 Howard Hughes Medical Institute, University of California, San Francisco, San Francisco, California, United States of America; Duke University Medical Center, United States of America

## Abstract

Survival at host temperature is a critical trait for pathogenic microbes of humans. Thermally dimorphic fungal pathogens, including *Histoplasma capsulatum*, are soil fungi that undergo dramatic changes in cell shape and virulence gene expression in response to host temperature. How these organisms link changes in temperature to both morphologic development and expression of virulence traits is unknown. Here we elucidate a temperature-responsive transcriptional network in *H. capsulatum*, which switches from a filamentous form in the environment to a pathogenic yeast form at body temperature. The circuit is driven by three highly conserved factors, Ryp1, Ryp2, and Ryp3, that are required for yeast-phase growth at 37°C. Ryp factors belong to distinct families of proteins that control developmental transitions in fungi: Ryp1 is a member of the WOPR family of transcription factors, and Ryp2 and Ryp3 are both members of the Velvet family of proteins whose molecular function is unknown. Here we provide the first evidence that these WOPR and Velvet proteins interact, and that Velvet proteins associate with DNA to drive gene expression. Using genome-wide chromatin immunoprecipitation studies, we determine that Ryp1, Ryp2, and Ryp3 associate with a large common set of genomic loci that includes known virulence genes, indicating that the Ryp factors directly control genes required for pathogenicity in addition to their role in regulating cell morphology. We further dissect the Ryp regulatory circuit by determining that a fourth transcription factor, which we name Ryp4, is required for yeast-phase growth and gene expression, associates with DNA, and displays interdependent regulation with Ryp1, Ryp2, and Ryp3. Finally, we define *cis*-acting motifs that recruit the Ryp factors to their interwoven network of temperature-responsive target genes. Taken together, our results reveal a positive feedback circuit that directs a broad transcriptional switch between environmental and pathogenic states in response to temperature.

## Abstract 2

Analysis of a transcriptional regulatory network in a fungal pathogen reveals that four interdependent transcription factors respond to human body temperature to trigger changes in cell shape and virulence gene expression.

## Author Summary

Microbial pathogens of humans display the ability to thrive at host temperature. So-called “thermally dimorphic” fungal pathogens, which include *Histoplasma capsulatum*, are a class of soil fungi that upon being inhaled into the human lung, undergo dramatic changes in cell shape and virulence gene expression in response to host temperature. The ability of these pathogens to cause disease is exquisitely coupled to temperature response. Here we elucidate the regulatory network that governs the ability of *H. capsulatum* to switch from a filamentous form in the soil environment to a pathogenic yeast form at body temperature. The circuit is driven by three transcription regulators (Ryp1, Ryp2, and Ryp3) that control yeast-phase growth. We show that these factors, which include two highly conserved proteins of the Velvet family of unknown function, bind to specific regulatory DNA elements and directly regulate expression of virulence genes. We identify and characterize Ryp4, a fourth regulator of this pathway, and define DNA motifs that recruit these transcription factors to their temperature-responsive target genes. Our results provide a molecular understanding of how changes in cell shape are linked to expression of virulence genes in thermally dimorphic fungi.

## Introduction

Cells adapt to their environment by responding to specific environmental stimuli such as light, temperature, and nutrients. For microbial pathogens, mammalian body temperature can signal the induction of pathways required for host colonization and pathogenesis [1]. One such group of organisms is the thermally dimorphic fungal pathogens, which include *Coccidioides*, *Paracoccidioides*, *Blastomyces*, and *Histoplasma* species. These evolutionarily related fungi are notable among fungal pathogens in that they all cause disease in healthy individuals [2]. Each of these organisms grows in a mold form in the soil, forming long, connected filaments that produce vegetative spores [3]. When the soil is aerosolized, filamentous cells and spores can be inhaled by mammalian hosts and converted into a parasitic form within the host lung. Conversion entails a dramatic change in cell shape to a budding yeast form for the majority of these pathogens, as well as the transcriptional induction of virulence genes required to cause disease in the host [3]. For all thermally dimorphic fungi, host temperature is the key signal that triggers this developmental switch, but little is known about the coordinated induction of morphologic changes and virulence gene expression by temperature.

*Histoplasma capsulatum*, which is endemic to the Ohio and Mississippi River Valleys of the United States, can cause life-threatening respiratory and/or systemic disease (histoplasmosis) [2],[4]. It is estimated that up to 25,000 people develop life-threatening infections in endemic regions each year, with at least 10-fold more mild or asymptomatic infections [2],[4]. Although the pathogen propagates as spores and in a filamentous form in the environment, *H. capsulatum* is found almost exclusively in the yeast form within mammalian hosts. Despite the prevalence of *H. capsulatum* and its threat to human health, we have a limited understanding of the transcriptional regulatory network that governs pathogenic yeast-phase growth. Previously, we identified three regulators, Ryp1, Ryp2, and Ryp3, and showed that they are required for yeast-phase growth [5],[6]. Whereas wild-type cells grow in the yeast form at 37°C, *ryp1*, *ryp2*, and *ryp3* mutants grow constitutively in the filamentous form independent of temperature. In wild-type cells, *RYP1*, *RYP2*, and *RYP3* transcripts and proteins accumulate preferentially at 37°C and each Ryp protein is required for the wild-type expression levels of the others [5],[6].

*RYP1* encodes a fungal-specific transcriptional regulator that is required for modifying the transcriptional program of *H. capsulatum* in response to temperature [5]. Ryp1 belongs to a conserved family of fungal proteins that regulate cellular differentiation in response to environmental signals. The best-studied member of this family of proteins is Wor1, which was identified as a master transcriptional regulator that controls a morphological switch required for mating in *Candida albicans* [7]–[9]. In the model yeast *Saccharomyces cerevisiae*, the Ryp1 ortholog, Mit1, is required for a morphologic switch that occurs under nutrient limitation [10]. Ryp1 orthologs in the plant pathogens *Fusarium oxysporum* (Sge1), *Fusarium graminerium* (Fgp1), and *Botrytis cinerae* (Reg1) are required for full pathogenicity and conidiation [11]–[13]. All of these observations signify the importance of Ryp1 orthologs for transduction of environmental cues to regulate cell morphology and virulence. Furthermore, it was recently demonstrated that Wor1 contains a DNA-binding domain that is conserved throughout the WOPR (Wor1, Pac2, Ryp1) family of proteins [14], suggesting that these regulators respond to specific signals by triggering a transcriptional program.

In contrast, Ryp2 and Ryp3 belong to the Velvet family of regulatory proteins [6], whose molecular function is unknown. This family is typified by Velvet A (VeA), which was initially characterized as a regulator of sexual spore production in *Aspergillus nidulans* [15],[16], but is now known to also regulate secondary metabolism and development in many fungi including *Aspergillus* species, *Fusarium* species, *Neurospora crassa*, and *Acremonium chrysogenum* (reviewed in [17]). In *H. capsulatum*, the VeA ortholog Vea1 has a role in sexual development but is dispensable for yeast-phase growth [18]. Additionally, many fungi have multiple Velvet family proteins that collaborate to serve regulatory functions. For example, in *A. nidulans*, three Velvet family proteins (the Ryp2 ortholog VosA, the Ryp3 ortholog VelB, and VeA itself) act together to regulate asexual and sexual development and secondary metabolism [19]. Notably, since Velvet family proteins do not contain canonical DNA binding domains or other domains of known function, their mechanistic role in regulation of developmental processes is unclear.

As noted above, both WOPR and Velvet family proteins are widely distributed among fungi, although the Hemiascomycetes, including *Saccharomyces* and *Candida* species, lack Velvet family proteins. Since both families of proteins are required for yeast-phase growth in *H. capsulatum*, we explored if and how these two distinct classes of fungal regulators work together to govern temperature-responsive traits by dissecting the Ryp regulatory network in *H. capsulatum*. To this end, we performed whole-genome transcriptional profiling and chromatin immunoprecipitation experiments to determine the shared and unique roles of Ryp1, Ryp2, and Ryp3 in regulating yeast-phase growth. We show that 96% of yeast-phase enriched transcripts are dependent on Ryp1, Ryp2, and Ryp3 for their enhanced expression in response to temperature, whereas 66% of filamentous-phase enriched transcripts require Ryp1, Ryp2, and Ryp3 to prevent their inappropriate expression at 37°C. We demonstrate that all three Ryp factors physically interact and associate with the upstream regions of a core set of target genes, including those required for yeast-phase growth and virulence. Additionally, we identify a fourth transcriptional regulator, Ryp4, to be a component of the Ryp regulatory network required for temperature-responsive yeast-phase growth. Finally, the identification of two distinct *cis*-acting regulatory sequences that are bound and utilized by Ryp proteins provides the first evidence that highly conserved Velvet family proteins can directly bind to DNA and activate gene expression using a unique *cis*-acting element. Overall, our results provide a molecular understanding of how regulation of cell morphology and virulence gene expression is coordinated in response to temperature in *H. capsulatum*.

## Results

### Ryp1, Ryp2, and Ryp3 Are Required for the Expression of Genes Associated With Growth in the Pathogenic Yeast Form

**Figure 1:**
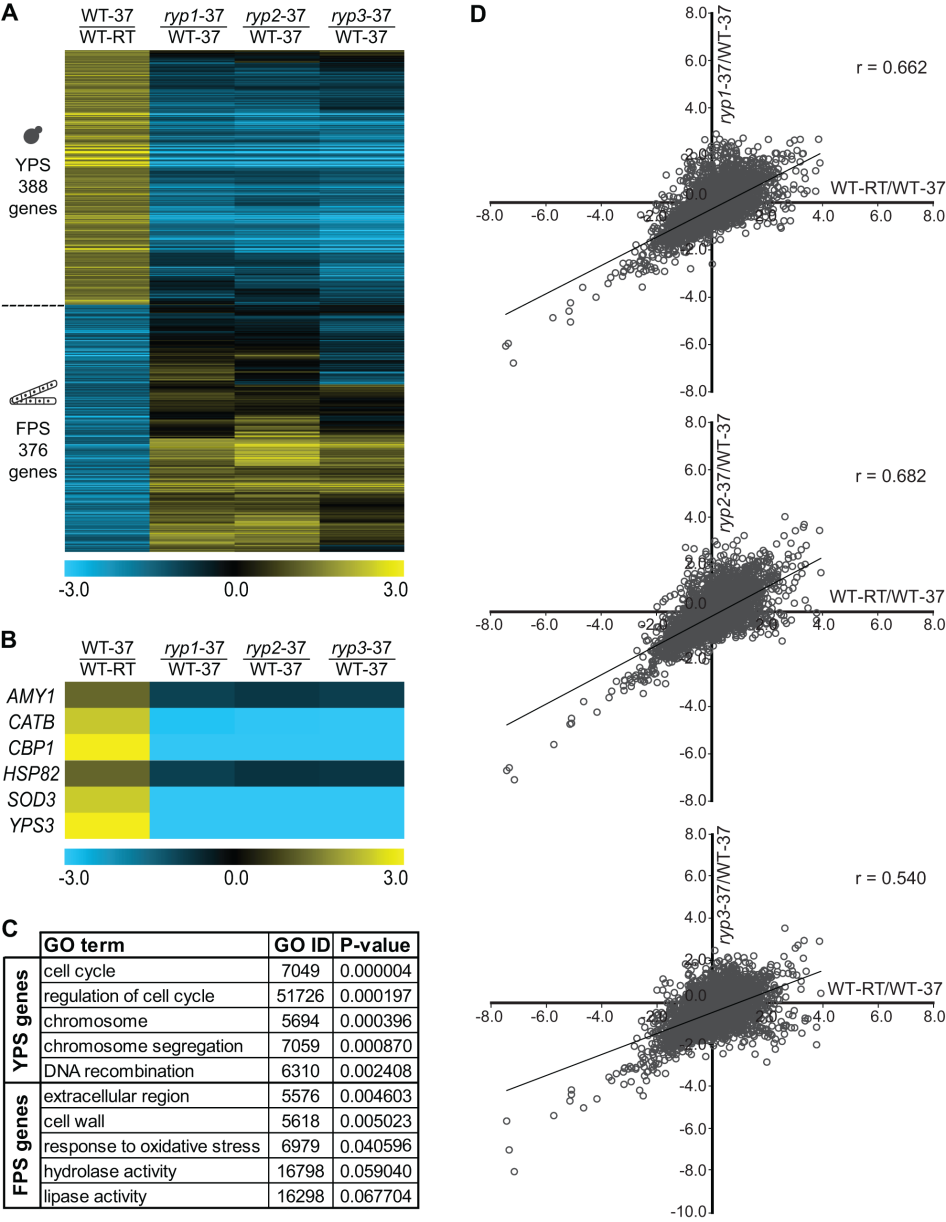
Ryp proteins are required for expression of yeast-phase specific genes. (A) Transcriptional profile comparisons of wild-type cells grown at 37°C (WT-37), wild-type cells grown at room temperature (WT-RT), and *ryp1*, *ryp2*, and *ryp3* mutants grown at 37°C represented as a heatmap. For a complete list of YPS and FPS genes see Table S1 (see Materials and Methods for bioinformatics analysis). (B) Select set of YPS genes involved in virulence is shown as a heatmap. Log2-based color scale is shown. (C) Phase-specific genes listed in Table S1 were subjected to analysis for GO term enrichment. Top five GO terms and enrichment *p* values are shown for both YPS and FPS genes. (D) Correlation between transcriptional profiles of YPS and FPS genes in *ryp* mutants grown at 37°C and wild-type cells grown at RT are presented as scatter plots with Pearson correlation coefficients (r).

Our previous studies showed that there are marked differences in the transcriptional profiles of wild-type yeast-form cells grown at 37°C and filamentous cells grown at room temperature [5],[20]. Cells lacking *RYP1*, *RYP2*, or *RYP3* grow constitutively as filaments independent of temperature [5],[6], and Ryp1 is required for the expression of the majority of the transcripts enriched during yeast-phase growth at 37°C [5]. Here we sought to understand whether Ryp2 and Ryp3 are also involved in regulating expression of genes required for yeast-phase growth. To this end, we performed whole-genome expression profiling experiments comparing the transcriptional profiles of multiple biological replicates of *ryp1*, *ryp2*, *ryp3* mutants and wild-type strains grown at room temperature (RT) and 37°C. We identified 388 genes with significantly increased transcript levels and 376 genes with significantly decreased transcript levels in wild-type yeast cells grown at 37°C compared to wild-type filaments grown at RT (Figure 1A and Table S1). These gene sets were referred to as yeast-phase–specific (YPS) and filamentous-phase–specific (FPS) genes, respectively.

Gene Ontology (GO) analysis showed that YPS genes were enriched in terms such as cell cycle regulation, chromosome segregation, and DNA recombination, all of which are characteristics of metabolically active, budding yeast cells (Figure 1C). In contrast, FPS genes were enriched in terms such as cell wall, extracellular region, response to oxidative stress, hydrolase, and lipase activity, consistent with the idea that filamentous cells have a distinct cell wall structure and produce enzymes related to saprophytic activity (Figure 1C). Additionally, YPS genes included the previously identified virulence genes of *H. capsulatum*: Genes encoding for calcium-binding protein 1 (*CBP1*) [21], yeast-phase specific protein 3 (*YPS3*) [22], super-oxide dismutase 3 (*SOD3*) [23], alpha-(1,4)-amylase (*AMY1*) [24], and heat-shock protein 90 (*HSP82*) [25] were among the most differentially regulated YPS genes. Furthermore, YPS genes included M antigen/catalase B (*CATB*), which has been shown to display yeast-specific expression in the *H. capsulatum* strain used here and encodes a secreted catalase that may help cope with oxidative stress in the host [26]. Transcript levels of these virulence genes were significantly down-regulated in *ryp* mutants, showing that Ryp factors are important for expression of virulence genes in *H. capsulatum* in response to temperature (Figure 1B).

Global comparison of the gene expression profile of wild-type cells to that of each *ryp* mutant revealed that the *ryp* mutants had a strongly diminished transcriptional response to temperature. The transcriptome of each *ryp* mutant grown at 37°C strongly resembled the transcriptome of wild-type cells grown at RT (Figure 1D). Additionally, transcriptional profiles of the individual *ryp* mutants were strikingly similar to each other (Figure 1A), indicating that they may act in the same temperature-responsive pathway. Transcript levels of the overwhelming majority of the YPS genes (96%) were decreased (>1.5-fold) in *ryp* mutants at 37°C, indicating that Ryp proteins were required for their wild-type expression level. Similarly, the Ryp proteins were required to prevent inappropriate expression of the majority of the FPS genes at host temperature: Transcript levels of 66% of the FPS genes were increased (>1.5-fold) in *ryp* mutants compared to the wild-type strain at 37°C. These results showed that Ryp1, Ryp2, and Ryp3 are master regulators required for the appropriate temperature-responsive transcriptional program in *H. capsulatum*.

As observed previously, our set of YPS genes included *RYP1*, *RYP2*, and *RYP3*, and each of the *RYPs* depended on the other two for its temperature-regulated expression (Figure S1B) [6]. Since *RYP* transcript levels are low at RT under laboratory conditions, we expected that the Ryp proteins might play only a minor role in regulation of gene expression at RT. Consistent with this idea, the Ryp factors are not required for the normal transcriptional profile of filaments grown at RT (Figure S1A). In sum, our transcriptional profiling experiments revealed that Ryp1, Ryp2, and Ryp3 are major regulators of yeast-phase growth at 37°C; are dispensable for filamentous-phase growth at RT; can either facilitate or repress transcript accumulation; and may act in the same pathway to regulate gene expression.

### Ryp1, Ryp2, and Ryp3 Associate with DNA Throughout the Genome

**Figure 2:**
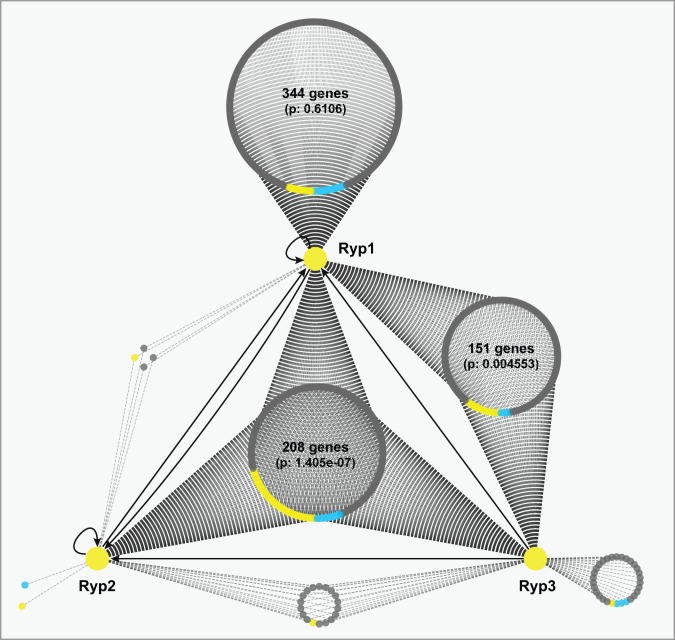
Ryp factors associate upstream of similar sets of genes. A network view of ChIP targets was generated using Cytoscape. Each large circle is composed of individual closed circles, each of which represents an individual target gene. YPS genes are colored in yellow, and FPS genes are colored in blue. The distribution of YPS and FPS genes in each category of ChIP targets (e.g., shared targets of Ryp1, 2, and 3) was compared to the whole genome (Figure S5) using the Wilcoxon test. The *p* values obtained in this analysis are shown. Additionally, targets shared by Ryp1 and Ryp3, as well as targets shared by all three Ryp factors, were enriched for YPS genes (*p* value <0.001) as determined by hypergeometric tests performed in R.

Previous studies reported that Wor1, an ortholog of Ryp1, associates with DNA at hundreds of intergenic regions to regulate gene expression in *C. albicans* [14]. However, genome-wide DNA associations of Ryp1 have not been investigated in *H. capsulatum*. Additionally, Velvet family proteins do not contain a known DNA-binding domain and the ability of Ryp2 and Ryp3 orthologs to associate with DNA has not been explored. To establish the genome-wide association of Ryp factors with DNA and to distinguish between direct and indirect targets in the Ryp regulons, we performed chromatin immunoprecipitation-on-chip (ChIP-chip) using antibodies raised against Ryp1, Ryp2, and Ryp3. Experiments were performed in either wild-type yeast cells grown at 37°C, or in the respective *ryp* mutant control grown at 37°C. We observed 361 ChIP events throughout the genome of wild-type cells (Figure 2, Figure S2A, and Table S2). Most notably, there were a large number of targets (182 loci) that associated with at least two Ryp factors, and 94 loci associated with all three Ryp factors, suggesting that Ryp1, Ryp2, and Ryp3 can act together to regulate gene expression. Interestingly, only Ryp1 had a large number of events (161 loci) that were not shared with other Ryp factors, indicating that Ryp1 has a broader regulon than Ryp2 and Ryp3 (Figure S2A). Further characterization of the ChIP events revealed that intergenic lengths corresponding to Ryp association events were significantly longer than the average intergenic length in the genome (Figure S2B). A similar trend was noted previously for Wor1 association events in *C. albicans*, but its biological significance is unknown [27]. Notably, shared ChIP events that involved all three Ryp factors showed the most drastic shift in intergenic length distribution.

**Figure 3:**
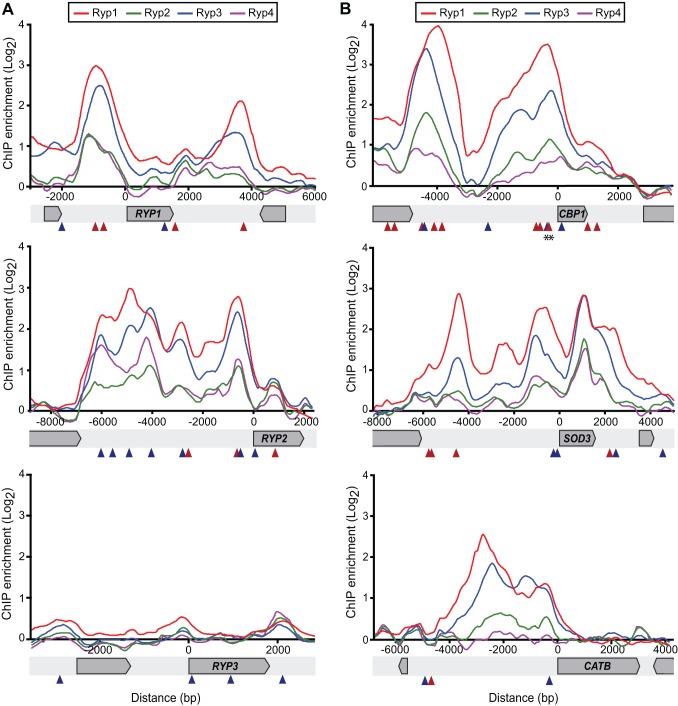
Ryp factors associate upstream of genes regulating morphology and virulence. ChIP-chip enrichment ratio versus genome coordinate for Ryp1, Ryp2, Ryp3, and Ryp4 ChIP events were plotted using Mochiview. Genome coordinates were adjusted to the start of the ORF of interest. Motif A (red triangles) and Motif B (blue triangles) locations shown in these loci were identified using MAST. Genomic regions of *RYP* genes (A) and virulence genes (B) are shown. Asterisks denote Motif A and Motif B probes used in the mobility shift assays in Figure 6C and 6D.

To map the genomic regions defined by ChIP-chip events to specific genes, we used a validated gene set that was defined previously based on gene expression and sequence conservation [28] and identified genes that lie downstream of each ChIP event (Table S2). Our first notable observation was that Ryp1, Ryp2, and Ryp3 showed interdependent regulation. All three Ryp factors associated upstream of *RYP1* and *RYP2*, although none of the three factors associated upstream of *RYP3* (Figure 3A and Table S2). To further explore the relationship between DNA association and gene expression, we overlaid gene expression data onto all ChIP-chip target genes (Figure 2). Targets that associated only with Ryp1 were not significantly enriched in YPS genes compared to the whole genome. In contrast, we observed significant enrichment of YPS genes for DNA-association events that were shared by Ryp1, Ryp2, and Ryp3, as well as events that were shared only by Ryp1 and Ryp3. This analysis revealed a correlation between shared association events and genes whose enhanced expression was induced by growth at host temperature. Strikingly, many of the known virulence genes (*CBP1*, *SOD3*, and *YPS3*) were shared Ryp targets (Figure 3B and Table S2). These results indicate that the known core virulence genes are direct targets of the Ryp factors, and suggest that the remaining shared Ryp targets are interesting candidates for potential virulence factors. Notably, shared Ryp targets and all Ryp targets were enriched for predicted signal peptides (*p* = 1.4e-06 and *p* = 2.0e-06, respectively) compared to the whole genome, which is of interest since secreted proteins produced by intracellular pathogens are often involved in virulence.

### Ryp1, Ryp2, and Ryp3 Regulate a Fourth Transcriptional Regulator, Ryp4, Which Is Required for Growth in the Pathogenic Yeast Form

**Figure 4:**
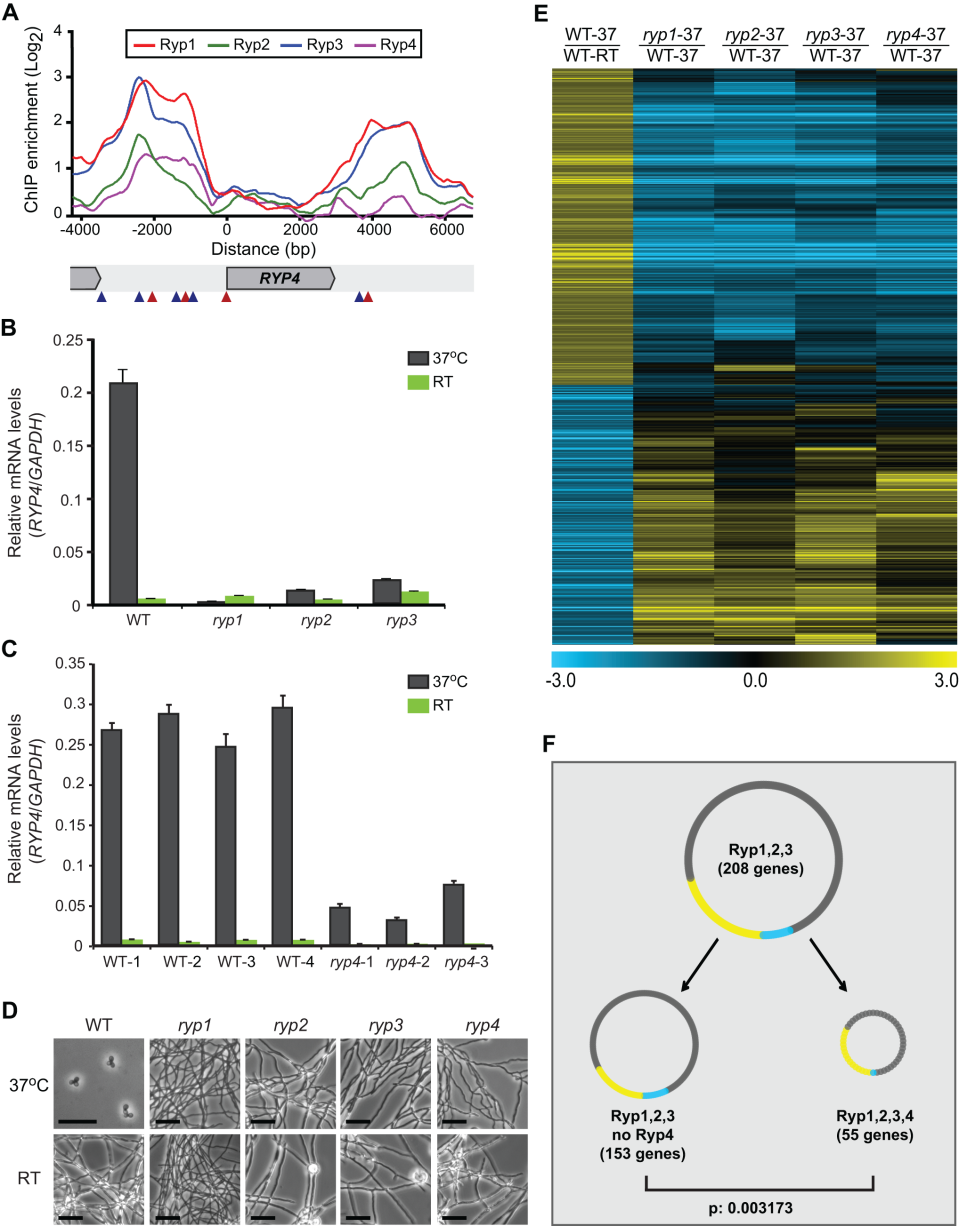
Ryp4 is required for yeast-phase growth. (A) ChIP-chip enrichment ratio versus genome coordinate for Ryp1, Ryp2, Ryp3, and Ryp4 ChIP events are shown at the *RYP4* locus. Genome coordinates were adjusted to the start of the ORF. Motif A (red triangles) and Motif B (blue triangles) locations shown in these loci were identified using MAST. (B, C) qRT-PCR was used to quantify relative levels of *RYP4* transcript in (B) *ryp1*, *ryp2*, *ryp3* insertion mutants and wild-type and (C) *ryp4* knockdown mutants and wild-type controls grown at 37°C and RT. *GAPDH* was used as a normalizer gene. qRT-PCRs were performed with at least two biological replicates for each strain. Triplicate measurements of representative replicates are graphed as mean ± standard deviation. (D) Phase-contrast microscopy images of *ryp1*, *ryp2*, *ryp3*, and *ryp4* mutants and wild-type controls grown at 37°C and RT are shown. Black bar equals 20 µm. (E) Transcriptional profile comparisons of wild-type grown at 37°C (WT_37), wild-type grown at room temperature (WT_RT), and *ryp1*, *ryp2*, *ryp3*, and *ryp4* mutants grown at 37°C represented as a heatmap. Phase-specific (YPS and FPS) genes are listed in Table S3 (see Materials and Methods for bioinformatics analysis). (F) Different categories of ChIP targets are shown as circles. Ryp1, Ryp2, and Ryp3 shared targets were split into two categories depending on the presence or absence of a Ryp4 ChIP event. The distribution of YPS and FPS genes in each category was compared to each other using the Wilcoxon test. The *p* value obtained in this analysis is shown. In this comparison, Ryp1, Ryp2, Ryp3, and Ryp4 shared targets are further enriched for YPS genes (*p* value <0.001) as determined by hypergeometric tests performed in R.

In addition to virulence factors, the shared Ryp targets included a single transcription factor with a known DNA-binding domain and yeast-phase–specific expression. The corresponding gene, designated HISTO_DM.Contig933.eannot.1650.final_new, encodes a Zn(II)_2_Cys_6_ zinc binuclear cluster domain protein (Figure 4A). As shown by microarray and qRT-PCR experiments, transcript levels of this gene were 6.5- to 35-fold higher at 37°C compared to RT, indicating that it displays enhanced expression in wild-type yeast-phase cells as compared to filaments (Figure 4B and Table S1). Moreover, Ryp1, Ryp2, and Ryp3 were required for expression of this gene at 37°C (Figure 4B and Table S1). We investigated the possibility that, similar to the *RYP* factors, HISTO_DM.Contig933.eannot.1650.final_new is essential for growth in the pathogenic yeast form. Since targeted gene disruption in *H. capsulatum* is inefficient, we generated knockdown strains using RNA interference (RNAi). We were able to deplete mRNA levels of HISTO_DM.Contig933.eannot.1650.final_new by 72%–88% (Figure 4C). Additionally, these RNAi strains were unable to grow in the yeast form and instead exhibited robust filamentous growth at 37°C, similar to the *ryp* mutants (Figure 4D). Loss of RNAi plasmids resulted in reversion to yeast-phase growth at 37°C (Figure S3A), indicating that the phenotype was dependent on the RNAi plasmids. Thus, we renamed this gene *RYP4* (Required for Yeast-Phase Growth). Although these morphologic studies revealed that *ryp4* knockdown strains appeared drastically different from wild-type cells grown at 37°C, they were indistinguishable from wild-type filaments in appearance when grown at RT (Figure 4D).

BLASTP analysis indicated that the closest homolog of Ryp4 is FacB of *A. nidulans* (E-value <1e-180), and phylogenetic analysis (see Materials and Methods) revealed that Ryp4 is an ortholog of FacB (Figure S3B and S3C). FacB is a transcriptional regulator of genes involved in acetate utilization in *Aspergillus* species and *N. crassa* [29]–[33]. Considering this conserved role in multiple fungal species, we investigated whether Ryp4 has a role in acetate utilization in *H. capsulatum*. However, unlike *facB* mutants in other organisms, *ryp4* knockdown strains were able to grow in acetate as a major carbon source (unpublished data), leading us to favor the hypothesis that Ryp4 has been rewired to regulate morphology in *H. capsulatum*.

### Ryp4 Is a Regulator of Genes Involved in Morphology and Virulence

To assess whether Ryp4 is required for normal yeast-phase–specific gene expression in response to temperature, we performed whole-genome expression profiling experiments using wild-type cells carrying control vectors (hereafter referred to as wild-type) and *ryp1*, *ryp2*, *ryp3*, and *ryp4* knockdown strains grown either at RT or at 37°C. In contrast to the gene expression studies performed in Figure 1 using insertion mutants, here we used *ryp1*, *ryp2*, and *ryp3* knockdown strains to match the *ryp4* knockdown strain (hereafter referred to as “mutant”). Statistical analyses revealed 441 YPS genes and 362 FPS genes in this dataset (Table S3). Similar to the aforementioned microarray dataset, regulation of the expression of the majority of YPS and FPS genes was dependent on Ryp1, Ryp2, Ryp3, and Ryp4 (Figure 4E and Table S3). Notably, the transcriptional profile of the *ryp4* mutant was very similar to that of *ryp1*, *ryp2*, and *ryp3* mutants (Figure 4E). The transcriptional profile of *ryp4* mutants grown at 37°C was similar to that of wild-type filamentous cells grown at RT (Figure S4A). Additionally, both microarray analysis and qRT-PCR experiments showed that Ryp4 was required for the expression of *RYP1*, *RYP2*, and *RYP3* (Figure S3D). In contrast to its critical role at 37°C, Ryp4 was not required for the normal transcriptional profile of filaments grown at RT (Figure S4B). Overall, these results indicate that Ryp4, like Ryp1, Ryp2, and Ryp3, is critical for temperature-regulated gene expression at 37°C.

To further explore the Ryp4 regulon, we performed ChIP-chip experiments in wild-type cells using antibodies raised against Ryp4. We identified 61 Ryp4 association events that occur in cells grown at 37°C (Table S2). Interestingly, the majority (74%) of these events were shared with other Ryp factors, indicating that Ryp4 acts in concert with other Ryp factors to regulate gene expression. Next, we identified genes downstream of each Ryp4 ChIP event (Table S2), and visualized these data together with Ryp1, Ryp2, and Ryp3 events (Figure S5). We found that about a quarter (26%) of Ryp1, Ryp2, and Ryp3 shared targets were also occupied by Ryp4 (Figure 4F). Furthermore, these common Ryp targets were even more significantly enriched for YPS genes than targets that were shared by Ryp1, Ryp2, and Ryp3, but not Ryp4 (Figure 4F). These YPS genes included core regulators of morphology (*RYP1*, *RYP2*, and *RYP4*) and genes required for virulence (*CBP1* and *SOD3*), further emphasizing the role of Ryp4 as a fundamental regulator of yeast-phase growth and an essential component of the temperature-responsive Ryp regulatory network (Figures 3A,B and 4A).

### Ryp1, Ryp2, and Ryp3 Physically Interact

**Figure 5:**
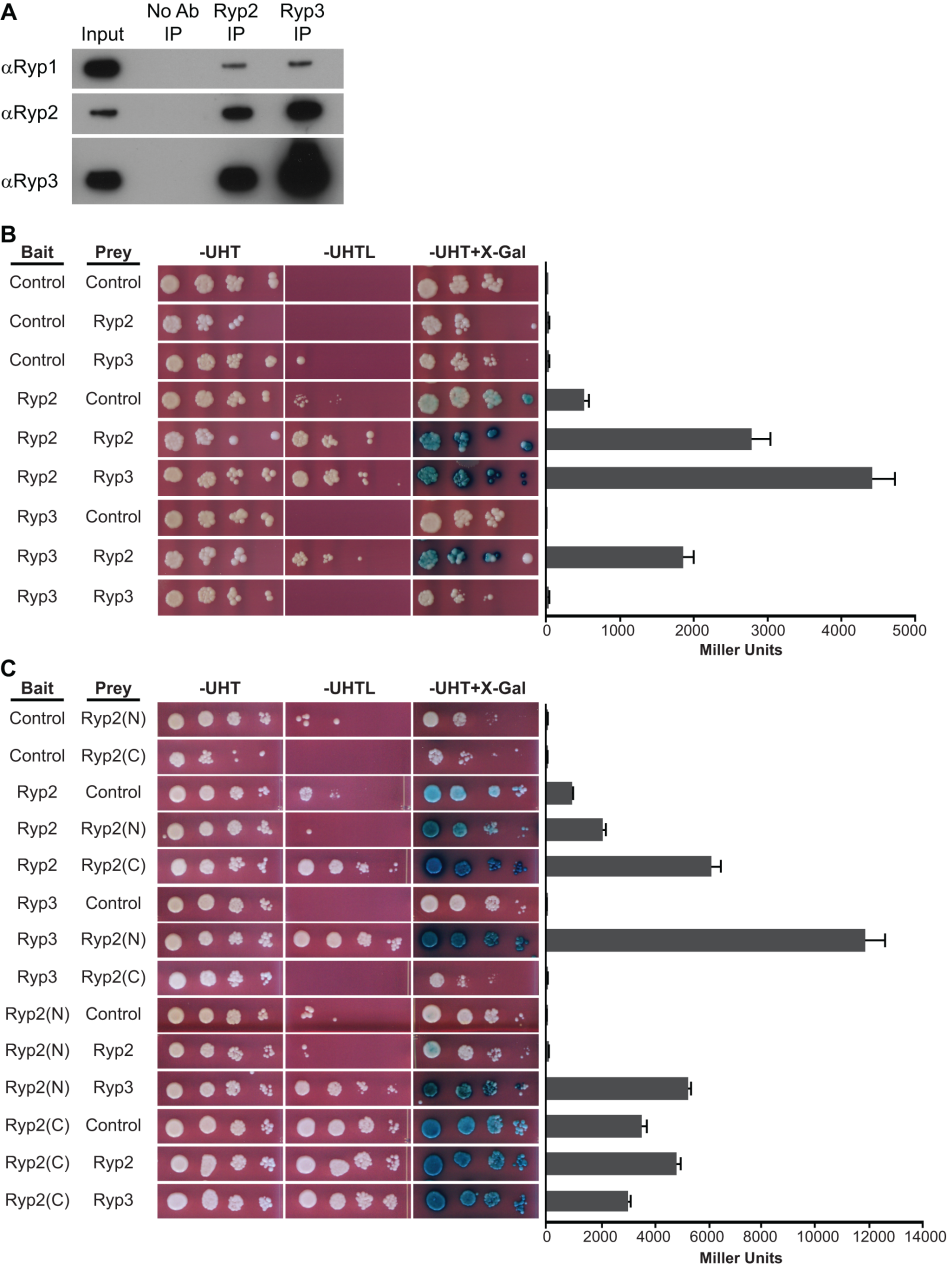
Ryp1, Ryp2, and Ryp3 physically interact. (A) Co-IP experiments were performed in wild-type cells grown at 37°C using Ryp2 and Ryp3 antibodies. Immunoblot shows detection of Ryp1, Ryp2, and Ryp3 in the input and elution fractions of Ryp2 and Ryp3 IPs. (B, C) Yeast-two-hybrid matings were performed with bait and prey strains listed. Diploid cells were spotted onto minimal media plates: -UHT, -Uracil/-Histidine/-Tryptophan; –UHTL, -Uracil/-Histidine/-Tryptophan/-Leucine. Growth on -UHTL plates and blue color on -UHT+X-gal plates indicate an interaction between proteins expressed in bait and prey strains. β-galactosidase activities were measured for three independent isolates of each strain. Quadruplicate measurements of representative isolates are graphed as mean ± standard deviation.

The ChIP data described above revealed that Ryp1, Ryp2, Ryp3, and Ryp4 share a large number of overlapping targets, suggesting that these proteins may physically interact. To test this hypothesis, we performed co-immunoprecipitation (co-IP) experiments in wild-type cells grown at 37°C using each of the Ryp antibodies. Of all possible co-IP combinations, we were able to reliably and reproducibly detect Ryp1, Ryp2, and Ryp3 in Ryp2 and Ryp3 IPs, indicating that at least these three Ryp proteins physically interact (Figure 5A). Ryp4 IPs did not yield reproducible interactions with other Ryp proteins. No Ryp protein signal was present in control IPs performed in the corresponding *ryp* mutant grown at 37°C (unpublished data).

In addition to these biochemical experiments, we used yeast two-hybrid experiments to assess Ryp1–Ryp2–Ryp3–Ryp4 interactions. We observed a reciprocal interaction between Ryp2 and Ryp3, confirming the co-IP results (Figure 5B). Our results also revealed that the Ryp2 N-terminus (which contains the Velvet domain), but not the Ryp2 C-terminus, is required for interaction with Ryp3 (Figure 5C). On the other hand, the Ryp2 C-terminus mediates Ryp2–Ryp2 interactions (Figure 5B and 5C). No interactions were observed for Ryp4 (unpublished data). Despite numerous attempts at transformation and analysis of multiple clones, we were unable to express Ryp1 bait or prey fusion proteins in the *S. cerevisiae* strains used for yeast-two-hybrid analysis. As a result, yeast-two-hybrid interactions with Ryp1 could not be assessed. Similarly, a previous study reported difficulty expressing yeast-two-hybrid constructs made with the Ryp1 ortholog from *F. oxysporum* [13], suggesting that expression of these Ryp1 two-hybrid fusion proteins is toxic in standard laboratory strains of *S. cerevisiae*.

### Ryp ChIP Events Are Enriched for Distinct *cis*-Acting Regulatory Sequences

**Figure 6:**
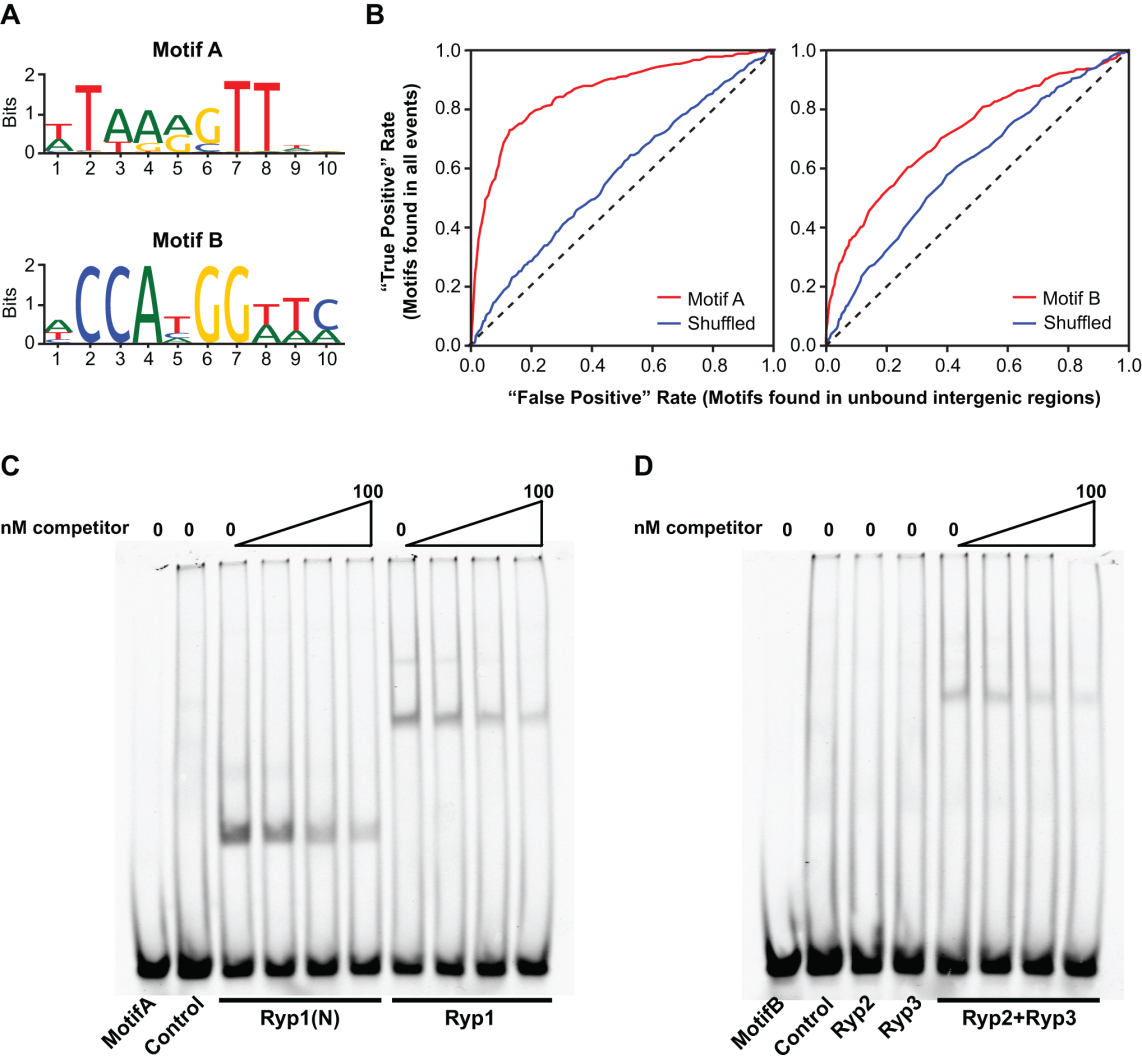
Ryp factors bind to specific *cis*-acting regulatory elements. (A) Motif A and Motif B logos are shown. (B) Motif specificity of Motif A, Motif B, and their shuffled versions were analyzed by ROC plots. True positive rate was defined as motifs found in all ChIP events. False positive rate was defined as motifs found in unbound intergenic regions. (C, D) His-tagged Ryp proteins were generated by a coupled *in vitro* transcription-translation reaction in wheat germ extracts, and purified using Ni-NTA beads. Extracts with no template DNA were processed similarly and included as controls. 60-bp fragments from the *CBP1* promoter were used as Motif A and Motif B probes (Figure 3B). 1 nM labeled probe and 2 µg of purified protein were used for each binding reaction. Binding to labeled Motif A or Motif B probes was competed by adding unlabeled probe in 10-fold, 50-fold, and 100-fold molar excess of the labeled probe.

To further explore the Ryp regulatory network, we analyzed each Ryp ChIP event set for the nonrandom occurrence of conserved *cis*-acting regulatory sequences (hereafter referred to as DNA motifs). This *de novo* motif analysis was especially interesting for Ryp2 and Ryp3, since there has been no prior evidence that Velvet family proteins associate with DNA, and thus, no DNA motifs have been identified for Velvet family proteins. Through the analysis of Ryp1, Ryp2, and Ryp3 ChIP events, we identified two distinct DNA motifs, which we named Motif A and Motif B. Specifically, different variants of Motifs A and B were identified through the analysis of Ryp ChIP events (Table S4, Figure S6A and S6B), and motifs that had the best predictive characteristics using receiver operating characteristic (ROC) plots (as described in [34]) were selected as Motif A and Motif B (Figure 6A). Motif A is very similar to the Wor1 motif, which was previously identified using biochemical approaches [14]. Identification of Motif A by a completely independent approach validates our ChIP-chip data and our motif analysis pipeline. Motif B, which is quite distinct from Motif A, did not resemble any previously identified motifs according to searches of the motif databases JASPAR (http://jaspar.genereg.net/) and YETFASCO (http://yetfasco.ccbr.utoronto.ca/). ROC plots using all ChIP events from each of the regulators demonstrated that Motif A and B were enriched in the entire ChIP set. Shuffled versions of each motif resulted in loss of specificity (Figure 6B). Of note, motif-finding algorithms did not yield a meaningful result for Ryp4, which is not surprising as the complex binding sites of zinc binuclear cluster transcriptional regulators can be difficult to predict [35],[36].

Further analysis revealed that Motif A was associated with Ryp1 ChIP events, whereas Motif B was associated with Ryp2 and Ryp3 ChIP events: Motif A specificity was dependent only on the inclusion of Ryp1 ChIP events (Figure S6C), whereas Motif B specificity was dependent only on the inclusion of Ryp2 and Ryp3 events (Figure S6D). Furthermore, Motif A enrichment was similar in all Ryp1 targets regardless of whether they were shared targets with the other Ryp factors or whether they associated only with Ryp1 (Figure S6E). In contrast, Motif B was enriched in Ryp1 targets that were shared with Ryp2 and Ryp3, but had no specificity in targets that were unique to Ryp1 (Figure S6E). These results corroborate the model that association of Ryp1 with DNA correlates with the presence of Motif A, and association of Ryp2 and Ryp3 with DNA correlates with the presence of Motif B.

Motifs A and B are distributed throughout the *H. capsulatum* genome and are present in many of the Ryp targets (Tables S5). Specific examples are shown in Figures 3 and 4 where Motif A and B distribution is shown in the upstream regulatory regions of *RYP1*, *RYP2*, *RYP4*, *CBP1*, *SOD3*, and *CATB*. Neither motif was found in the *RYP3* upstream region (Figure 3), which is in agreement with our ChIP results that the Ryp factors do not associate upstream of *RYP3*.

### Ryp Factors Bind to Distinct DNA Sequences

Our motif analyses suggested that Ryp1 associates with DNA via Motif A, whereas Velvet family proteins (Ryp2 and Ryp3) associate with DNA via Motif B. To test whether Ryp1, Ryp2, or Ryp3 can directly bind to Motif A or Motif B, we performed electrophoretic mobility shift assays (EMSAs). The promoter region of the *CBP1* gene, which associates with the Ryp proteins by ChIP-chip and contains both Motif A and Motif B (Figure 3B), was used to design two distinct 60 bp probes encompassing either Motif A or Motif B (Figure 3B). His-tagged versions of Ryp1, Ryp2, and Ryp3 were synthesized in coupled *in vitro* transcription and translation reactions and then subjected to purification. Additionally, it was already known that the N-terminus of *C. albicans* Wor1, which is highly homologous to the N-terminus of Ryp1, contains two globular domains that are sufficient to bind the Wor1 motif [14]. Therefore, we also synthesized and purified a His-tagged version of the N-terminus of Ryp1 (Ryp1(N), 1–267 aa) that harbors only the globular domains.

EMSA revealed that full-length Ryp1 binds directly to Motif A, whereas control extracts contained no binding activity (Figure 6C). In analogy to Wor1, we also observed that Ryp1(N) binds directly to Motif A (Figure 6C). To explore the ability of Ryp2 and Ryp3 to bind Motif B, we performed mobility shift assays with either Velvet protein alone or with a combination of Ryp2 and Ryp3. Whereas mobility shift assays performed with either Ryp2 or Ryp3 showed no binding activity, addition of both of these Velvet proteins resulted in binding of the Motif B probe (Figure 6D). All observed band shifts were diminished upon addition of unlabeled competitor probe into the binding reactions. Notably, these experiments provide the first evidence that Velvet family proteins bind DNA directly.

### Ryp Factors Are Necessary and Sufficient to Drive Gene Expression Via Motifs A and B

Since Ryp1 is sufficient to bind Motif A and Ryp2–Ryp3 is sufficient to bind Motif B, we next used an *in vivo* transcriptional activation assay [14] to explore whether each motif was sufficient to drive gene expression in a heterologous system when the appropriate Ryp proteins were expressed. We cloned a single copy of Motif A or B upstream of the UAS-less *CYC1* promoter fused to the *lacZ* gene. We introduced these reporter plasmids along with constructs expressing the Ryp proteins, either individually or in combination, into *S. cerevisiae* and monitored β-galactosidase activity. *S. cerevisiae* provides an ideal heterologous expression system for our experiments, since the reporter strain we used lacks Ryp1 orthologs, and Velvet family proteins are not present in *S. cerevisiae*.

**Figure 7:**
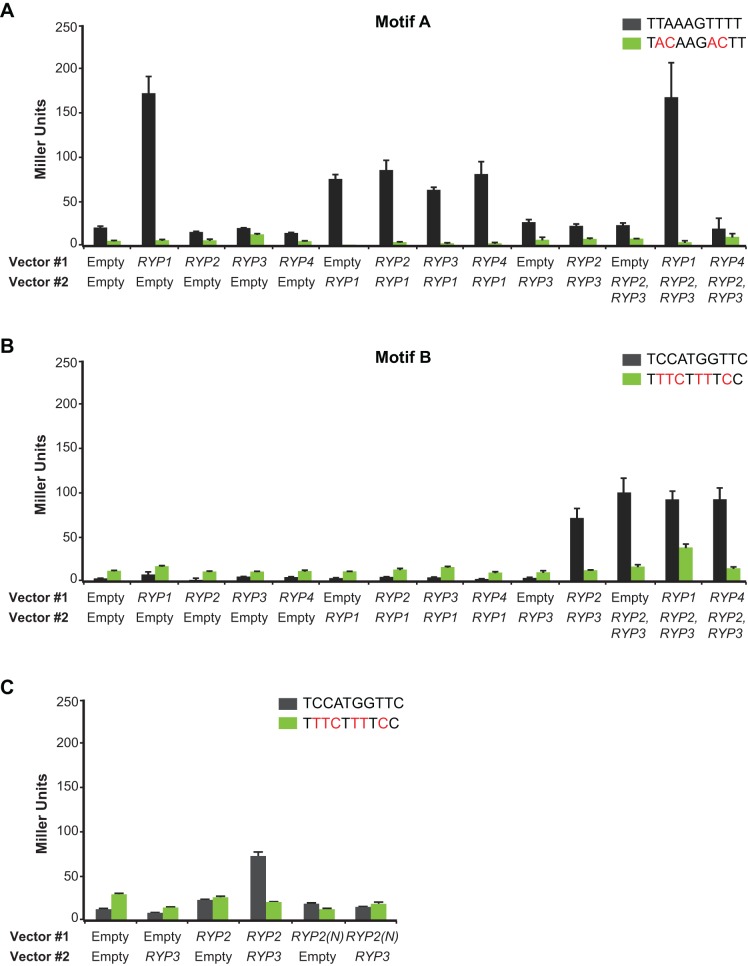
Ryp factors can drive gene expression using specific *cis*-acting regulatory elements. (A) Motif A (B, C) Motif B, and their mutated versions were cloned into UAS-less *CYC1* promoter fused to *lacZ* gene. Point mutations made in the motifs are shown in red. *RYP* genes were cloned into two different vectors with two different markers. Each of these vectors were transformed into motif-containing yeast strains as labeled. β-galactosidase activities were measured for three independent isolates of each strain. Quadruplicate measurements of representative isolates are graphed as mean ± standard deviation.

In the strains that contained the Motif A reporter construct, β-galactosidase activity was detected only when Ryp1 was heterologously expressed (Figure 7A). This activity was severely diminished when the conserved residues of Motif A were mutated. Co-expression of Ryp2, Ryp3, or Ryp4 individually with Ryp1, or Ryp1, Ryp2, and Ryp3 together, did not lead to an increase in β-galactosidase activity (Figure 7A). These results indicate that Ryp1 is necessary and sufficient to drive gene expression via Motif A. In contrast, in strains containing Motif B, β-galactosidase activity was detected only when Ryp2 and Ryp3 were expressed together, but not when Ryp2 or Ryp3 were expressed singly (Figure 7B). This activity was also dependent on the conserved nucleotides of Motif B. Co-expression of Ryp1 or Ryp4 along with Ryp2 and Ryp3 did not enhance Ryp2-Ryp3–dependent β-galactosidase activity (Figure 7B). Additionally, even though the Ryp2 N-terminus (Ryp2(N)), which contains the Velvet domain, is sufficient to interact with Ryp3 (Figure 5C), co-expression of Ryp2(N) and Ryp3 was not sufficient to drive gene expression using Motif B (Figure 7C). For all these experiments, β-galactosidase activity was dependent on the presence of the appropriate motif (Figure S7A), but independent of motif orientation (Figure S7B and S7C). These results indicate that Ryp2 and Ryp3 together are necessary and sufficient to drive gene expression via Motif B and, taken together with the EMSA studies, provide the first evidence that Velvet proteins bind DNA via a conserved motif to direct transcriptional activation.

## Discussion

Cells have derived complex regulatory networks to reprogram their transcriptional response upon changes in the environment. In thermally dimorphic fungi, regulation of cell morphology and virulence traits is coupled: they respond to host temperature by altering their cell shape and inducing virulence gene expression. This study is the first elucidation of the transcriptional circuitry underlying this dramatic change in any of these organisms. In this article, we have shown that an interlocking network of transcription factors regulate each other and common target genes to trigger a transcriptional program that is required for cell shape changes and virulence gene expression in response to host temperature in *H. capsulatum*.

**Figure 8:**
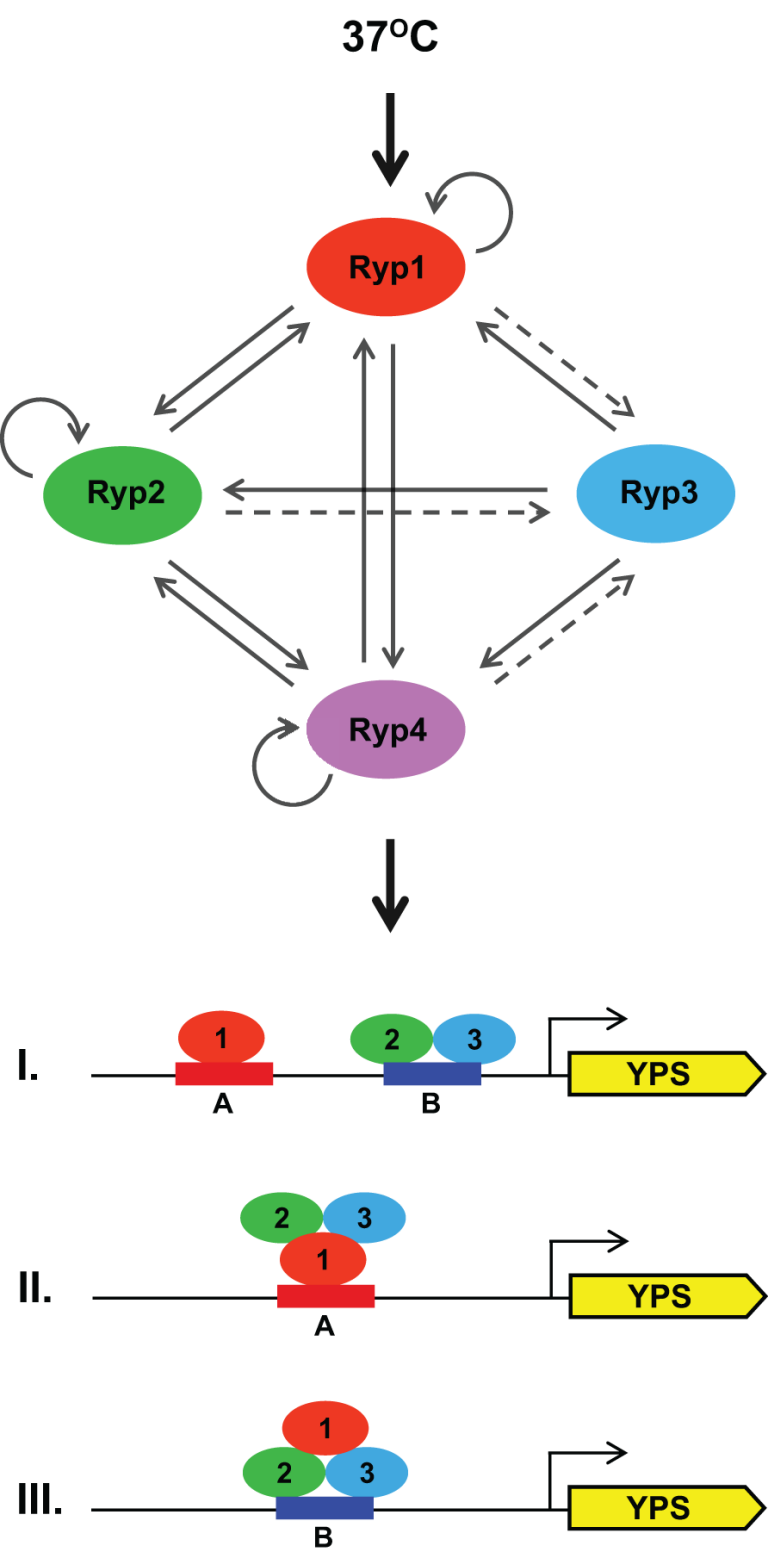
Ryp proteins are involved in a temperature-responsive complex transcriptional network. A model for the temperature-responsive Ryp network is shown. ChIP-chip interactions are depicted by solid arrows and indirect regulation is depicted by a dashed arrow. All four Ryp proteins are expressed at increased levels at 37°C, require each of the others for their expression, and associate with the promoter regions of *RYP1*, *RYP2*, and *RYP4*. Even though none of the Ryp proteins associate with *RYP3* promoter, *RYP3* expression levels depend on the other Ryp proteins. *De novo* motif finding approaches and *in vivo* transcriptional activation assays reveal that Ryp1 is necessary and sufficient to drive gene expression using Motif A, and Ryp2-Ryp3 co-expression is necessary and sufficient to drive gene expression using Motif B. We hypothesize that YPS gene expression in response to growth at host temperature can be achieved through many possible combinations of motifs and Ryp factor association. Three such scenarios are shown.

Each of the Ryp transcription factors described here, including the newly discovered Ryp4, is absolutely required for the normal transcriptional profile of cells grown at 37°C. The structure of the corresponding transcription factor network is diagrammed in Figure 8, which illustrates the connectivity between the four proteins required to program the switch from filamentous to yeast-form growth in response to temperature. Each Ryp factor is required for the expression of the others, and each associates upstream of the *RYP1*, *RYP2*, and *RYP4* genes—but none of the factors, including Ryp3 itself, associates upstream of the *RYP3* gene. Thus we propose that at 37°C, each of the factors acts in a positive-feedback loop to regulate itself and each of the others. The exception is Ryp3, which regulates the other factors but is only indirectly regulated by them and does not directly regulate itself. Perhaps the interlocking nature of the network promotes a robust response by amplifying and stabilizing the signal generated by increased temperature. Additionally, the absolute requirement for activation of all four Ryp factors may provide specificity by requiring a sustained increase in temperature (as experienced within a mammalian host) to trigger the appropriate developmental program. Moreover, the requirement for multiple factors may allow unknown host signals other than temperature to influence the transition from filaments to yeast-form cells. The nature of the molecular signal that induces an initial increase in Ryp expression in response to temperature is unknown, but others have identified a histidine kinase, *DRK1*, that is required for yeast-phase growth in *H. capsulatum* and the related thermal dimorph *Blastomyces dermatitidis* [37]. A possible relationship between *DRK1* and regulation of Ryp factors has not been explored.

Ryp factors are required both to promote transcription of genes with enhanced expression at 37°C and to prevent the inappropriate expression of filament-associated genes at this temperature. Transcriptional profiling of wild-type cells grown either at 37°C (in the yeast form) or at room temperature (in the filamentous form) yielded 764 genes (about 8.5% of the genome) that passed our criteria for differential expression in one of the two growth phases. Remarkably, 96% of YPS genes required each of the four Ryp proteins to achieve differential expression in response to temperature. The majority of this regulon is a consequence of indirect regulation by the Ryp factors, since only a fraction of YPS genes are downstream of Ryp association events. The most significant enrichment for YPS genes was observed for shared targets of multiple Ryp factors: about 21% of shared targets were YPS genes as opposed to 4% of the whole genome. Interestingly, Ryp association events that are unique to an individual Ryp factor, such as the large cohort of ChIP-chip events that associate only with Ryp1 and not the other three Ryp factors, showed no enrichment for YPS genes. Additionally, Ryp targets that were not YPS genes did not depend on Ryp factors for their expression under standard laboratory conditions. It is possible that Ryp factors are poised upstream of these genes to regulate their expression in response to environmental signals other than temperature, such as light intensity, nutrient availability or exposure to reactive radicals in the host.

Recruitment of the Ryp transcription factors to their direct targets is driven, at least in part, by DNA sequence motifs that are sufficient to recruit either Ryp1 (Motif A) or the Ryp2–Ryp3 complex (Motif B) (Figure 8). Motif A, which is highly similar to the DNA motif that was defined for the Ryp1 ortholog Wor1, is enriched in Ryp1 targets, whereas Motif B is enriched in Ryp2 and Ryp3 targets. Figure 8 illustrates the major categories of motif distribution for YPS gene regulatory regions that associate with Ryp factors. Interestingly, some YPS genes that contain only Motif A or only Motif B show association with Ryp1, Ryp2, and Ryp3. Although there are several models that could explain this multifactorial association, we favor the idea that there are different subcomplexes of Ryp proteins in the cell. For example, we propose that Ryp1 associates with DNA directly via Motif A in the absence of Ryp2 and Ryp3, thereby accounting for the many Ryp1 ChIP-chip events that are not shared by the other Ryp factors. Supporting this model is the finding that Ryp1 is sufficient to drive gene expression via Motif A in *S. cerevisiae*, and the biochemical experiments that show that the Ryp1 ortholog Wor1 associates directly with DNA via a motif that is highly similar to Motif A [14]. Alternatively, Ryp1 can be present in a complex with Ryp2 and Ryp3, and then could be recruited to the DNA either directly, via Motif A, or indirectly, via interaction of Ryp2–Ryp3 with Motif B. This model also predicts that Ryp2–Ryp3 can be recruited to the DNA either directly or via interaction with Ryp1. Interestingly, the existence of ChIP-chip targets that contain Motif A but associate only with Ryp1 and not with Ryp2–Ryp3 suggests that there is a genomic feature, such as chromosomal context for Motif A, that distinguishes the set of Ryp1-only targets from the shared targets. For example, perhaps chromosomal context causes the association of Ryp1 with Motif A in the shared targets to require nonspecific interactions of Ryp2–Ryp3 with the DNA. Interestingly, we identified a number of ChIP-chip targets that associate only with Ryp3, and found that these targets are enriched for Motif B. Since binding of Ryp3 to Motif B, at least *in vitro*, requires the presence of Ryp2, it is possible that these orphan events are actually shared with Ryp2 but fall below our level of detection for Ryp2 association. Alternatively, Ryp3 might be recruited to these targets via association with one of the other two Velvet domain proteins in *H. capsulatum*. Finally, the newly identified yeast-phase regulator Ryp4 seems to play no role in recognition of Motif A or Motif B, at least *in vitro*, and is incapable of driving gene expression via these motifs in a heterologous transcriptional activation assay. Additionally, we did not observe any physical interactions between Ryp4 and other Ryp factors. Taken together, these observations suggest that a third *cis*-regulatory element might recruit Ryp4 to its targets.

In this study, we provide the first evidence that Velvet family proteins such as Ryp2 and Ryp3 can bind DNA directly. This family of proteins is well studied in environmental fungi, fungal plant pathogens, and an opportunistic fungal pathogen of humans; however, although physical interactions between multiple Velvet family proteins have been defined, the molecular mechanism of Velvet family proteins in general is unknown. In addition to showing binding of Ryp2 and Ryp3 to the DNA via mobility shift assays, we show that Motif B is sufficient to drive gene expression in the model yeast *S. cerevisiae* when Ryp2 and Ryp3 are co-expressed. The N-terminus of Ryp2, which contains the Velvet domain, is required for Ryp2–Ryp3 interaction in the yeast two-hybrid assay, whereas the C-terminus of Ryp2 mediates multimerization of Ryp2 (Figure 5C). In a previous study, the C-terminal region of the Ryp2 ortholog VosA was predicted to be a transcriptional activation domain [38]. Since co-expression of the Ryp2 N-terminus with Ryp3 is not sufficient to activate gene expression through Motif B (Figure 7C), we conclude that the Ryp2 C-terminus is required for activation of gene expression in our *S. cerevisiae* transcriptional activation assays. Whether a Ryp2 homodimer or multimer has a role in gene expression in *H. capsulatum* or is important for the higher order formation of a Ryp complex remains to be investigated.

One of the most interesting findings to arise from this network analysis is that the Ryp factors, which themselves are YPS genes that are required for morphology, directly regulate YPS genes that are required for pathogenesis (e.g., *CBP1*, *SOD3*, and *YPS3*). A hallmark of thermally dimorphic fungal pathogens is that temperature regulates cell shape and other factors required for pathogenesis. In the case of *H. capsulatum*, yeast-phase morphology is thought to be critical for the lifestyle of this fungus in the host, especially since replication of the fungus within host immune cells is likely to be incompatible with filamentous growth. Thus, Ryp factors catalyze both the cell shape change and the increased expression of known virulence factors, resulting in a molecular link between temperature and the expression of traits required to cause disease in a mammalian host. A link between morphology and virulence has been explored for many of the major human fungal pathogens, perhaps most extensively for *C. albicans* (reviewed in [39]). Unlike the thermally dimorphic fungi, which are found almost exclusively in a hyphal form in the soil and a yeast or spherule form in the host, *C. albicans* is present in multiple morphologies in the host, and filamentous cells are thought to play a major role in disease. We observed co-regulation of yeast-phase morphology and known virulence genes in *H. capsulatum*. Similarly, numerous studies in *C. albicans* have observed that hyphal formation is coordinated with the expression of genes required for adhesion, host tissue invasion, antifungal drug resistance, and oxidative stress response [39], suggesting that a subset of fungal pathogens have evolved regulatory circuits that link virulence gene expression with morphologic changes. Notably, despite the importance of the yeast-to-hyphal transition in the infectivity of *C. albicans*, a systematic analysis of a large-scale *C. albicans* deletion library showed that morphology can be uncoupled from virulence. Although a large number of mutants were identified to have defects in both virulence and morphology, a sizeable number of mutants were defective for virulence despite maintaining normal morphologic responses *in vitro*, and some mutants displayed normal infectivity despite having substantial defects in morphogenesis [40]. In contrast, the ability to differentiate into yeast-form cells is likely to be essential for the intracellular parasitism that is characteristic of *H. capsulatum* infection. Therefore, it may be the case that yeast-phase morphology is categorically linked to virulence in the thermally dimorphic fungal pathogens.

Finally, this work expands the rich history of elucidating fungal transcription circuits to understand regulatory networks in eukaryotic cells. Studies of fungal gene regulation continue to provide examples of novel DNA-binding domains, as was previously the case with the Ryp1 family of proteins [14]. In this study, we show association between DNA and two Velvet family proteins that lack known DNA-binding domains, now implicating this highly conserved protein family in direct gene regulation. The most interesting molecular implication of these data is that the Velvet domain could be a novel DNA binding domain. Additionally, this analysis reveals a fundamental example of transcriptional rewiring: although Ryp4 regulates acetate utilization genes in related fungi outside of the thermal dimorphs, in *H. capsulatum* its major role is to regulate the transcriptional response to temperature. As a result, the temperature-dependent circuit elucidated here demonstrates cooperation and regulation between three distinct families of transcription factors: WOPR, Velvet, and Zn(II)_2_Cys_6_. Expression of each of the Ryp proteins has been wired to be absolutely dependent on all of the others, so strains that lack any of the regulators fail to undergo a developmental program in response to temperature. This network structure is interesting, in part because of the regulatory implications described above, but also because the relationship between orthologs of these transcription factors is not static throughout the fungal kingdom. For example, Hemiascomycetes have retained WOPR family members but lack Velvet family proteins. These phylogenetic differences provide an opportunity to study how regulatory circuits evolve. Finally, the role of the Ryp proteins in the life cycle of other thermally dimorphic fungi has not been examined. Elucidating the nature of this circuit in these related fungal pathogens will provide insight on the evolution of this temperature-responsive circuit.

## Materials and Methods

### Strains and Culture Conditions

*H. capsulatum* strains G217B (ATCC26032) and G217B*ura5* (WU15) were kind gifts from Dr. William Goldman (University of North Carolina, Chapel Hill). *ryp1*, *ryp2*, *ryp3* T-DNA, and knockdown mutants were previously generated [5],[6]. *ryp4* knockdown mutants and wild-type strains with control RNAi plasmids were generated in this study.

#### Yeast-phase cultures

All cultures were grown in *Histoplasma* macrophage medium (HMM) broth or on HMM agarose plates. G217B*ura5* cultures were supplemented with 0.2 mg/ml uracil. Liquid cultures were grown at 37°C with 5% CO_2_ and shaking at 150 rpm and were passaged in every 2–3 d with 1∶25 dilution into fresh medium. For transcriptional profiling experiments, liquid cultures were grown at 120 rpm to minimize growth condition differences with filamentous-phase cultures. Plates were grown at 37°C with 5% CO_2_.

#### Filamentous-phase cultures

As previously described [5], yeast cultures were converted to filamentous-phase by plating a heavy inoculum of yeast cells onto Sabouraud Dextrose agar (Difco) and incubating at 25°C for ∼2 wk. *ryp* mutants taken from frozen stock were also plated on Sabouraud Dextrose agar and incubated either at 37°C with 5% CO_2_ or at 25°C. The resulting filamentous cells were inoculated into HMM and grown for 2 wk at 37°C with 5% CO_2_ or room temperature with shaking at 120 rpm to establish a filamentous-phase culture. Cultures were passaged with 1∶ 5 dilution into fresh medium every 1–3 wk.

### DNA Manipulations

All plasmids and primers used in this study are listed in Table S6 and Table S7, respectively. All plasmids were maintained in *Escherichia coli* DH5α strain and sequenced to ensure no mutation was introduced during the cloning process.

#### Construction of RNAi plasmids

Two ∼500 bp fragments of the *RYP4* ORF were amplified from G217B gDNA using OAS2235-36 and OAS2237-38. Using Gateway Cloning Technology (Life Technologies-Invitrogen), these two fragments were cloned into pDONR/Zeo vector, generating pSB7 and pSB8 to target two different regions in the *RYP4* mRNA. Combining pFANTAi4 with donor vectors generated integrating RNAi plasmids (pSB18 and pSB19). pSB23 is a Gateway-compatible destination plasmid and was derived from pCR186 (a kind gift from Dr. Chad Rappleye, The Ohio State University). Combining pSB23 with donor vectors (pSB7 and pSB8) generated episomal RNAi plasmids (pSB30 and pSB31).

#### Construction of yeast-two-hybrid plasmids

*S. cerevisiae* strains EGY48/pSH18-34 and W303A, and plasmids pEG202 and pJSC401 were kind gifts from Dr. Jeffery S. Cox (University of California, San Francisco). The *RYP3* ORF was amplified from G217B cDNA using OAS3415-3551, digested with EcoRI and XhoI, and ligated into similarly digested bait and prey plasmids pEG202 and pJSC401, respectively. This process generated the *RYP3* bait plasmid (pSB74) and the *RYP3* prey plasmid (pSB80). pSB74 was transformed into the EGY48/pSH18-34 strain and pSB80 was transformed into W303A using standard protocols [41]. Due to the unavailability of compatible restriction enzyme sites, the *RYP2* ORF was cloned directly into *S. cerevisiae* strains using standard protocols [41]. *RYP2* full-length, *RYP2*-N-terminal (1–624 bp), and *RYP2*-C-terminal (595–1215 bp) fragments were amplified from G217B cDNA using OAS3559-60, OAS3559-96, and OAS3595-60, respectively. These three fragments were re-amplified using OAS3565-66 to increase homologous flanking sites to 50 bp. Extended fragments were cloned into linearized pEG202 generating the *RYP2* full-length (pSB73), *RYP2*-N-terminal (pSB86), and *RYP2*-C-terminal (pSB87) bait plasmids. To generate prey plasmids, *RYP2* full-length, *RYP2*-N-terminal (1–624 bp), and *RYP2*-C-terminal (595–1215 bp) fragments were amplified from G217B cDNA using OAS3567-68, OAS3567-4032, and OAS4031-3568, respectively; and these fragments were re-amplified using OAS3573-74. Extended fragments were cloned into linearized pJSC401 generating the *RYP2* full-length (pSB79), *RYP2*-N-terminal (pJW1), and *RYP2*-C-terminal (pJW2) prey plasmids. Plasmids that were constructed in *S. cerevisiae* strains were rescued from yeast and transformed into *E. coli* DH5α strains for further manipulations.

#### Construction of plasmids used for expression and purification of Ryp proteins

For expression of *RYP* genes, *RYP1*-N-terminus (1–801 bp), *RYP1*, *RYP2*, and *RYP3* ORFs were amplified from G217B cDNA using OAS4110-11, OAS4114-15, OAS4118-19, and OAS4122-24, respectively. These reactions were cleaned and used as a template for the following amplification reaction. The goal of the second amplification was to introduce the T7 promoter, β-globin, and Kozak sequences to the 5′ end, and sequences encoding the TEV cleavage site, 6XHis-tag, polyA tail, and T7 terminator sequences to the 3′ end of each *RYP* fragment. To this end, all *RYP* fragments were amplified for 10 cycles in the presence of low amounts (0.05 µM) of OAS4106-07. Then, 0.5 µM of OAS4102-03 was added to the reactions and an additional 20 cycles of PCR amplifications were done. Resulting fragments were purified using QIAquick PCR purification kit (Qiagen) and cloned into pCR2.1-TOPO vector using the TOPO TA cloning kit (Invitrogen), generating pSB122 (*RYP1*-N-terminal), pSB124 (*RYP1*), pSB128 (*RYP2*), and pSB130 (*RYP3*) plasmids.

#### Construction of plasmids used in the *in vivo* transcriptional assay

For expression of *RYP* genes, *RYP1*, *RYP2*, *RYP3*, and *RYP4* ORFs, as well as *RYP2* N-terminal fragments, were amplified from G217B cDNA using OAS3411-3549, OAS3555-3750, OAS3415-3551, OAS3751-52, and OAS3555-4205, respectively. *RYP1*, *RYP2*, *RYP3*, *RYP4*, and *RYP2* N-terminal fragments were digested with EcoRI-XhoI, BamHI-SalI, EcoRI-XhoI, and SpeI-SalI, and BamHI-SalI, respectively, and ligated into similarly digested p413TEF and p414TEF vectors. Resulting plasmids are listed in Table S6. For the expression of *RYP2* and *RYP3* on the same plasmid, P*_TEF1_*-*RYP2*-t*_CYC1_* fragment was amplified from pSB95 using OAS3783-84. This fragment was digested with SacI and ligated into SacI-digested pSB97, resulting in pSB115. To generate P*_CYC1_*_ΔUAS_-*lacZ* plasmids with DNA motifs, primer pairs OAS3893-94, OAS3895-96, OAS3901-2, and OAS3103-4 were annealed, digested with XhoI, and ligated into XhoI-digested P*_CYC1_*_ΔUAS_-*lacZ* plasmid. DNA sequencing was used to identify plasmids with single insertion and correct orientation of motifs. Resulting plasmids are listed in Table S6. All plasmids were transformed into *S. cerevisiae* Δ*mit1*Δ*yhr177w* strain, which was a kind gift from Dr. Alexander D. Johnson (University of California, San Francisco).

### Generation of *ryp4* Knockdown Strains

Introduction of integrating and episomal RNAi plasmids into *H. capsulatum* G217B*ura5* strain was done as previously described [6]. *ryp4* knockdown strains with integrated RNAi constructs were used in all experiments, except for the plasmid loss experiments where episomal RNAi plasmids were used.

### Plasmid Loss Experiments

Four independent *ryp4* mutants generated with two different episomal RNAi plasmids (pSB30 and pSB31) were grown in HMM broth supplemented with uracil (0.2 mg/ml) at 37°C, 120 rpm with 5% CO_2_. After 4 wk, an inoculating loop was used to transfer cells from each flask onto HMM+uracil agarose plates followed by incubation at 37°C with 5% CO_2_. Individual yeast-phase colonies were streaked again and tested for uracil auxotrophy on HMM agarose plates. Representative images of the resulting strains are shown in Figure S3A.

### Microscopy

All images were obtained using a Zeiss Axiovert 200 microscope with 40× Phase objective.

### Quantitative Reverse Transcription PCR (qRT-PCR)

Total RNA from *H. capsulatum* strains was harvested using Trizol (Life Technologies-Invitrogen) following manufacturer's instructions. RNA was treated with RNase-free DNase set (Qiagen) and cleaned using the RNeasy mini kit (Qiagen). 2 µg of total RNA was used in cDNA synthesis using Superscript RT II (Life Technologies-Invitrogen) following manufacturer's instructions. cDNAs were used to amplify *RYP1*, *2*, *3*, *4*, and *GADPH* transcripts using OAS1057-58, OAS1942-43, OAS1944-45, OAS3320-31, and OAS1452-53, respectively (Table S7). qRT-PCR reactions were performed in the Stratagene Mx3000P QPCR System (Agilent Technologies) and contained 300 nm of each primer and 0.8× FastStart Universal SYBR Green Master mix (Roche). The transcript levels of *RYP* genes were normalized to the *GAPDH* transcript levels.

### Whole-Genome Transcriptional Profiling

Total RNA from *H. capsulatum* strains was harvested as previously described [5]. Reference RNA was generated by mixing total RNA from *ryp* mutants and wild-type control strains in three different ratios: For microarray experiments with *ryp* T-DNA mutants, equal amounts of total RNA from each *ryp* mutant and wild-type were combined. For microarray experiments with *ryp1*, *ryp2*, and *ryp3* knockdown strains, total RNA from wild-type yeast, wild-type filaments, and *ryp* knockdown strains were mixed in a 1∶1∶2 ratio. For microarray experiments with *ryp4* knockdown strains, total RNA from wild-type yeast, wild-type filaments, and *ryp4* knockdown strains was mixed in 1∶1∶1 ratio. For each sample, 15 µg of total RNA was used to generate amino-allyl–labeled cDNA as described previously [42] with the following modifications: Reverse transcription reaction was performed with Superscript RT II (Life Technologies-Invitrogen), and reactions were cleaned using QIAquick PCR clean-up kit (Qiagen) with phosphate wash buffer (5 mM KPO_4_, pH 8.0, 80% ethanol). cDNA from each *ryp* mutants and wild-type controls was labeled with Cy5 (GE Healthcare Life Sciences-Amersham) as described [42] and competitively hybridized against the Cy3-labeled pooled reference sample using a *H. capsulatum* whole-genome 70-mer oligonucleotide microarray. For the experiments with *ryp* T-DNA mutants, there were four to six replicates for each strain and condition, and for the experiments with *ryp* knockdown strains, there were three to 12 replicates for each strain and condition. Raw data for all hybridizations (total of 88) performed are available through Gene Expression Omnibus (GEO, www.ncbi.nlm.nih.gov/geo/) with accession numbers GSE46936, GSE46937, GSE46938, and GSE46939 (GEO superseries accession number GSE 47832).

### Whole-Genome Transcriptional Profiling Data Analysis

Arrays were scanned on a GenePix 4000B scanner (Molecular Devices) to determine the intensity units in the 635- and 523-nm channels (detecting Cy5 and Cy3, respectively) for each spot on the microarray. Data were analyzed by using GENEPIX PRO version 6.0, NOMAD (http://nomad2.ucsf.edu/NOMAD/nomad-cgi/login.pl), and MultiExperiment Viewer 4.0 (www.tm4.org/mev.html) [43],[44]. After removal of values for flagged spots, background subtraction, and median normalization, the ratio of the median Cy5 intensity/median Cy3 intensity for these spots was used for further analysis. For the genes represented by multiple 70 mer probes, only values for the first probe were used for subsequent analysis. Normalized data was analyzed by Bayesian Analysis for Gene Expression Levels (BAGEL) to obtain relative expression levels for each spot in each condition or mutant [45]. Using the values obtained by BAGEL, we compared any two samples by dividing the value of the first sample by the value of the second sample. To determine the number and identity of genes that changed significantly in expression in any given comparison, we used >2.0-fold change in transcript levels and *p* value <0.01 as a cutoff criteria. Results of these analyses are given in Tables S1 and S3.

### Gene Ontology (GO) Analysis

A directed graph was constructed from all “is-a” relationships in the 12/7/2011 version of the Gene Ontology (go_daily-termdb.obo-xml.gz downloaded from the GO Consortium website [http://www.geneontology.org/]). GO-to-gene edges were added from the 12/7/2011 versions of the AspGD [http://www.aspgd.org/] [46], CGD [http://www.candidagenome.org/] [47], and SGD [http://www.yeastgenome.org/] [48] GO association files for *A. nidulans* (An), *A. fumigatus* (Af), *C. albicans* (Ca), *Candida glabrata* (Cg), and *S. cerevisiae* (Sc). Gene-to-gene edges were added for An/Af/Ca/Cg/Sc genes to their *H. capsulatum* G217B (HcG217B) orthologs based on InParanoid mapping of each genome pair (using default parameters with no outgroup). InterPro domains (IPR) from the Pfam, TIGRFAMS, SMART, PANTHER, and Gene3D databases from InterPro version 34 were mapped to HcG217B genes with InterProScan version 4.8 [49]. GO-to-IPR and IPR-to-gene edges were added to the graph by parsing the InterProScan results. An/Af/Ca/Cg/Sc genes with no ortholog in HcG217B were pruned from the graph, as were obsolete GO terms. The set of G217B genes deriving from each GO term was tabulated by traversing the graph in reverse topological sort order, assigning each parent node to the union of the G217B genes spanned by its children. For each query gene set, the GO association graph was pruned to the subset of GO terms spanning the query. GO terms in the resulting subgraph with a single GO term parent and child were considered uninformative relative to the query and were replaced by a direct edge from parent to child. We further removed any GO terms that were associated only with a single gene. The graph was further reduced to only GO terms present in the union of the AspGD/CGD/SGD GO slim sets (12/7/2011 version). For each GO term spanning k genes in the query, the probability of that term spanning at least k terms in a random gene set of the same size was calculated from the hypergeometric distribution as in [50]. The probability calculation was carried out using the phyper function in R [51] with graph operations implemented using NetworkX [52]. The top five terms are reported for each query with no adjustment for multiple hypothesis testing.

### Ryp4 Orthology Searches

Initial sets of Ryp4 homologs were identified using HMMer 3.0 and the Pfam hidden Markov models (HMMs) for the fungal Zn(II)_2_Cys_6_ binuclear cluster domain (PF00172.13) and the fungal-specific transcription factor domain (PF04082.13). Each HMM was searched against the BROAD predicted gene sets for *Coccidioides immitis* RS, *Trichophyton verrucosum* HKI 0517, *Paracoccidioides brasiliensis* Pb03, *Blastomyces dermatitidis* er-3, *Fusarium graminearum* ph-1, *Neurospora crassa* OR74A, *Magnaporthe oryzae* 70-15, *Botrytis cinerea* B05.10, and *Stagnospora nodorum* SN15; the SGD curated gene set for *Saccharomyces cerevisiae* S288C; the AspGD curated gene set for *Aspergillus nidulans* FGSC; and the GSC predicted gene set for *Histoplasma capsulatum* G217B using hmmscan. Hits were aligned to each HMM using hmmalign, and neighbor-joining trees were generated from the aligned domain sequences using CLUSTALW 2.1. For PF00172.13, Ryp4 was found in a monophyletic clade, sister to Sip4, with orthologs from each target species except for *B. cinerea*. For PF04082.13, Ryp4 was found in a monophyletic clade identical to the previous one except for the gain of *B. cinerea* BC1G_13551 and the loss of Sc Cat8. TBLASTN of the Ryp4 protein sequence against the *B. cinerea* genome yielded a hit to the Zn(II)_2_Cys_6_ domain (E = 2e-29) about 170 bp upstream of BC1G_13551, consistent with an incorrectly predicted 5′ for this gene. Based on these results, the union of pezizomycotina hits was annotated as orthologs of Ryp4. The full-length protein sequences of the Ryp4 orthologs, as well as Cat8 and Sip4, were aligned, and a bootstrapped (*n* = 1,000) neighbor-joining tree was generated using CLUSTALW.

### Chromatin Immunoprecipitation-on-Tiling Array (ChIP-Chip)

*ryp1*, *ryp2*, *ryp3*, and *ryp4* mutants and wild-type (G217B) cells were grown at 37°C. Cells were fixed and collected as previously described [5]. Frozen cell pellets were ground using a mortar and a pestle in liquid nitrogen or in Retsch Mixer Mill MM 400. Chromatin immunoprecipitation was performed as described [53] with the following modifications: 300 mg of ground samples were vortexed vigorously in lysis buffer [50 mM Hepes/KOH (pH 7.5), 140 mM NaCl, 1 mM EDTA, 1% Triton X-100, 0.1% sodium deoxycholate supplemented with Halt protease and phosphatase inhibitors (Pierce)] for 3 h with 0.5 mm glass beads to lyse the cells. Following cell lysis, lysates were sonicated in a Diagenode Bioruptor for 30 min (30 s on, 1 min off) to shear DNA. Input DNA was collected and the rest of the sample was subjected to immunoprecipitation. For filamentous-phase samples (*ryp* mutants), lysates from three identical tubes were combined prior to antibody incubation. Each sample was incubated overnight at 4°C with 20 µg of polyclonal antibodies against portion of Ryp1 (ID:2877, ELDKPFPPGEKKRA), Ryp2 (ID:237, QTNRDYPFYNGPDAKRPR), Ryp3 (ID:338, GIKIPIRKDGVKGPRGGQ), or Ryp4 (ID:8, PPPQQSLQGWSPEEW). On the next day, 40 µl of Protein-A Sepharose 4B Fast Flow (Sigma-Aldrich) beads were added to the lysate-antibody mixture and further incubated for 2 h. Subsequently, beads were collected by centrifugation and washed nine times. Protein–DNA complexes that are bound to the beads were collected by incubating beads at 65°C with elution buffer (50 mM Tris HCl pH 8.0, 10 mM EDTA, 1% SDS). Protein–DNA crosslinks in input and ChIPed samples were reversed overnight at 65°C. Proteins were digested with proteinase K and DNA was purified with phenol∶chloroform extraction as described [53]. Both input and output DNA were amplified and labeled with fluorescent dyes (Cy3 and Cy5) using strand displacement amplification following published protocols [53]. Labeled DNA samples were hybridized following Agilent's ChIP-on-chip protocol onto Agilent 2× 400K arrays comprised of 60 mer oligos tiling the entire *H. capsulatum* genome at a frequency of one probe per 50 bp. Oligos were selected based on a previously published method [54].

### ChIP-Chip Data Analysis

Slides were scanned with an Agilent scanner and raw ratios were obtained with the Agilent ChIP_107_Sep09 protocol. Background subtracted probe intensities were transformed to log2 (red/green) ratios (M) and log2 (sqrt(red*green)) geometric mean intensities (A), excluding probes with intensities below background. The M,A values were fit using the LOWESS algorithm [55] as implemented in R [51], and the fit curve was subtracted from the transformed data to yield lowess normalized log2 ratio values. Normalized spot intensities are available through GEO with accession number GSE47341 (GEO superseries accession number GSE 47832). After normalization, data were analyzed using Mochiview [56]. Data from three biological replicates for each Ryp-IP were analyzed together to identify peaks. ChIP-chip experiments done with *ryp* mutants were used as a negative control in this analysis. Default parameters of Mochiview peak extraction were used with the exception of increasing total random samples to 100,000 and maximum random samples to 100 (*p* value <0.001). Median+Interquartile range (IQR) was used as a threshold to filter extracted peaks. Peaks that were eliminated based on the noisy enrichment values in *ryp* mutants were included back if there was >2-fold difference between wild-type and *ryp* mutant enrichment ratios. Additionally, peaks that were greater than a median value for a given Ryp event and had another Ryp event greater than median+IQR were also included in the finalized list of events (Table S2). ChIP enrichment ratios plotted in Figures 3 and 4 were generated using ChIP tracks that were a combination of three biological replicates for each Ryp-IP.

Each ChIP event was mapped to specific genes using an *H. capsulatum* G217B strain validated gene set that was defined previously using gene expression and sequence conservation criteria [28]. Genes that have a ChIP event in their 5′ region within a 10 kb distance from the center of the peak were listed as target genes (Table S2). In addition to target genes found in the validated gene set, some additional target genes were included if they had detectable expression levels in the whole-genome transcriptional profiling experiments performed in this study.

### Signal Peptide Predictions

Signal peptide and transmembrane helix prediction algorithm Phobius 1.01 [57] was run with default parameters on the full G217B predicted protein set, excluding two sequences with predicted internal stops. Three nondisjoint prediction sets were derived from the Phobius output: genes predicted to have a signal sequence, genes predicted to have at least one transmembrane helix, and the intersection of the previous two sets. For each phobius prediction set spanning k genes in a given Ryp associated set, the probability of that term spanning at least k terms in a random gene set of the same size was calculated from the hypergeometric distribution as in [50]: P(k≤X) = 1−sum∧[k−1]_[i = 0] c(M,i)*c(N−M,n−i)/c(N,i), where c is the binomial coefficient function, N is the size of the full gene set, n is the size of the Ryp associated set, and M is the size of the phobius prediction set. The probability calculation was carried out using the phyper function in R [51].

### *De Novo* Motif Finding

Each set of Ryp ChIP events was randomly split into two sets using Mochiview. Each set was subjected to motif finding algorithms using Mochiview and Bioprospector [56],[58]. For each set, the top five motifs identified using Mochiview and the top three motifs identified using Bioprospector were further analyzed for specificity in the corresponding randomly split set. Motif searches were carried out using MAST [59] as implemented in version 4.8.1 of the MEME suite [60]. The MAST -hit_list parameter was used to yield all nonoverlapping motif instances in the query sequences with no adjustment for query length or number of motif hits (such that a reported *p* value reflects only the alignment of the motif instance to the query matrix). For ROC plots, the motif threshold (-mt) was set to 0.01 in order to explore a wide range of possible parameter values. For genome-wide searches, the motif threshold was set to the values determined from the ROC plots using false positive rate of 10% as a cutoff (corresponding to *p* values of 2.08e-04 for Motif A and 9.18e-05 for Motif B). Motif locations identified in the genome are given in Table S5.

### Expression and Purification of Ryp Proteins

For expression of the Ryp1-N-terminus, Ryp1, Ryp2, and Ryp3 proteins for EMSA experiments, we subjected pSB122, pSB124, pSB128, and pSB130, respectively, to TNT Coupled Wheat Germ Extract systems (Promega) following the manufacturer's instructions. For purification of Ryp proteins, eight 50-µl reactions were pooled and diluted 10-fold in binding buffer (10 mM Tris-HCl, pH 8.0, 50 mM KCl, 5% glycerol, 1% TritonX-100, and 20 mM imidazole) supplemented with HALT phosphatase and protease inhibitors (Pierce). For each sample, 100 µl of Ni-NTA agarose beads (Qiagen) was washed in binding buffer and incubated with extracts for 1 h at 4°C. Following the incubation, beads were washed once with binding buffer, and once with binding buffer with no detergents. His-tagged proteins were recovered with elution buffer (10 mM Tris-HCl, pH 8.0, 50 mM KCl, 5% glycerol, 250 mM imidazole). The presence of His-tagged Ryp proteins was confirmed by SDS-PAGE analysis and Western blotting. Wheat germ extract with no DNA template was subjected to a similar purification process and use as a control in mobility shift assays.

### EMSA

5′-IRDye800-labeled Motif A and Motif B probes were prepared by annealing 5′-IRDye800-CBP1-MotifA-Fwd and CBP1-MotifA-Rev, and 5′-IRDye800-CBP1-MotifB-Fwd and CBP1-MotifB-Rev, respectively, in 10 mM Tris-HCl, pH 7.9, 50 mM NaCl, 10 mM MgCl_2_, and 1 mM DTT. Nonlabeled competitor probes were prepared similarly with nonlabeled oligonucleotides (Table S7). Two µg of each purified protein (Ryp1-N-terminus, Ryp1, Ryp2, Ryp3, or control extract) and 1 nM of labeled probes were mixed in binding buffer (10 mM Tris-HCl, pH 8.0, 50 mM KCl, 5% glycerol, 1 mM EDTA, 0.5 mM DTT, 100 ug/ml BSA, and 25 ug/ml poly(dI:dC)) and incubated for 30 min at room temperature. Reactions were separated on 6% DNA retardation gels (Invitrogen) in 0.5× TBE buffer. Mobility shifts were visualized and analyzed using the ODYSSEY imaging system (LI-COR Biosciences).

### Co-immunoprecipitation

Wild-type (G217B) cells grown to late log phase at 37°C were harvested by filtration, and the pellet was frozen in liquid nitrogen. Whole cell extracts were made by cryogrinding the pellet in Retsch Mixer Mill MM 400. Co-immunoprecipitation experiments were performed using the Dynabeads Co-immunoprecipitation kit from Invitrogen following the manufacturer's instructions. Briefly, 100 ug of polyclonal α-Ryp2 (ID:387, SQSAGHMQSPSQVPPAWG) or α-Ryp3 (ID:356, SHGSKGQDGEGEDWENEG) antibodies were covalently linked to 5 mg of magnetic beads using Dynabeads Antibody Coupling kit. To prepare cell lysate, 2 g of ground samples were mixed with lysis buffer, vortexed, and spun down. Then, supernatant was incubated with 5 mg of antibody-coupled magnetic beads for 8 h at 4°C. After multiple washes, protein complexes that are bound to antibody-coupled beads were eluted in low pH buffer provided by the manufacturer. IPs with *ryp2* and *ryp3* mutants grown at 37°C and no antibody control were performed similarly. All fractions were separated by SDS-PAGE, visualized by silver staining, and analyzed by Western blotting using standard procedures. Polyclonal α-Ryp1 (ID:3873, ASSYQPGPPASMSWNTAATG), α-Ryp2 (ID:387, SQSAGHMQSPSQVPPAWG), or α-Ryp3 (ID:356, SHGSKGQDGEGEDWENEG) antibodies were used to detect Ryp proteins.

### β-Galactosidase Assays

β-galactosidase assays were performed as previously described [61]. At least three independent isolates of each *S. cerevisiae* strain were grown to late log (for the yeast-two-hybrid assay) or stationary (for the *in vivo* transcriptional activation assay) phase. Each isolate was assayed in quadruplicate, and the results of representative isolates are shown in Figures 5, 7, and S7.

## Supporting Information

Figure S1 Ryp factors are required for each other's expression. (A) Correlation between transcriptional profiles of YPS and FPS genes in *ryp* mutants and wild-type cells grown at room temperature are presented as scatter plots with Pearson correlation coefficients (r, *n* = 9,289). (B) qRT-PCR was used to quantify relative levels of *RYP1*, *RYP2*, *and RYP3* transcripts in *ryp1*, *ryp2*, and *ryp3* mutants and wild-type cells grown at 37°C and room temperature (RT). *GAPDH* was used as a normalizer gene. Experiments were performed with at least two biological replicates for each strain. Triplicate measurements of representative replicates are graphed as the mean ± standard deviation.
(TIF)

Figure S2 Ryp events occur at longer intergenic regions. (A) Numbers of Ryp1, Ryp2, and Ryp3 ChIP events are shown as a Venn diagram. (B) Intergenic length distributions for the whole genome (black line) and Ryp1, Ryp2, and Ryp3 individual and common ChIP events (red lines) are shown. The differences between length distributions were compared using the Wilcoxon test.
(TIF)

Figure S3 Ryp4 is required for yeast-phase growth and the expression of *RYP* genes. (A) Phase-contrast microscopy images of wild-type cells, or wild-type cells carrying either a vector control or *RYP4* RNAi plasmid are shown. RNAi vectors were maintained episomally and strains were grown at 37°C. *ryp4* knockdown strains were grown without selection to allow growth of clones that underwent spontaneous plasmid loss. Isolates of these strains that converted to the yeast form were analyzed to confirm that they had lost the RNAi marker. Black bar equals 20 µm. (B, C) Ryp4 is an ortholog of FacB. Initial sets of Ryp4 homologs were identified using HMMer 3.0 and the Pfam hidden Markov models (HMMs) for the fungal Zn(II)_2_Cys_6_ binuclear cluster domain (PF00172.13, shown in black boxes) and the fungal-specific transcription factor domain (PF04082.13, shown in grey boxes). Details of the databases used and searches are given in Materials and Methods. The full-length protein sequences of the Ryp4 orthologs, as well as Cat8 and Sip4, were aligned (C), and a bootstrapped (*n* = 1,000) neighbor-joining tree (B) was generated using CLUSTALW. (D) qRT-PCR was used to quantify relative levels of *RYP1*, *RYP2*, and *RYP3* transcripts in *ryp4* mutants and wild-type controls grown at 37°C and room temperature (RT). *GAPDH* was used as a normalizer gene. Experiments were performed with at least two biological replicates for each strain. Triplicate measurements of representative replicates are graphed as mean ± standard deviation.
(TIF)

Figure S4 Ryp knockdown mutants display similar transcriptional profiles to that of wild-type filaments. Correlation between transcriptional profiles of YPS and FPS genes in *ryp* mutants grown at (A) 37°C and (B) room temperature and wild-type cells grown at room temperature are presented as scatter plots with Pearson correlation coefficients (r, *n* = 9,289).
(TIF)

Figure S5 Ryp4 associates upstream of Ryp1, Ryp2, and Ryp3 targets. A network view of ChIP targets was generated using Cytoscape software. Each large open circle is composed of individual closed circles that represent individual genes. YPS genes are colored in yellow, and FPS genes are colored in blue. Microarrays used in this study represent 9,289 genes, which were also colored similarly as shown. The distribution of YPS and FPS genes in each type of ChIP event was compared to the whole genome using the Wilcoxon test. The *p* values obtained in this analysis are given. Additionally, Ryp1, Ryp3; Ryp1, Ryp2, Ryp3; and Ryp1, Ryp2, Ryp3, Ryp4 shared targets were enriched for YPS genes (*p* value <0.0001) as determined by hypergeometric tests performed in R.
(TIF)

Figure S6 Ryp events are enriched for specific DNA sequences. Logos of (A) Motif A and (B) Motif B variants are shown. (C, D) Motif specificity of (C) Motif A and (D) Motif B were analyzed by ROC plots. True positive rate was defined as motifs found in ChIP events that contained or excluded a given Ryp event. False positive rate was defined as motifs found in unbound intergenic regions. (E) Similarly, motif specificity of Motif A and Motif B was analyzed in Ryp1-only or shared events by ROC plots.
(TIF)

Figure S7 Motif A and Motif B are sufficient to drive gene expression in the presence of Ryp factors. *RYP* genes were cloned into two different vectors with two different markers. Each of these vectors were transformed into yeast strains with plasmids containing the UAS-less *CYC1* promoter fused to the *lacZ* gene to generate “no motif” controls (A). Additionally, complementary sequences of (B) Motif A, (C) Motif B, and their mutated versions were cloned into a plasmid containing the UAS-less *CYC1* promoter fused to the *lacZ* gene. Point mutations made in the motifs are shown in red. Each of the motif and Ryp plasmids were transformed into yeast strains as labeled. β-galactosidase activities were measured for three independent isolates of each strain. Quadruplicate measurements of representative isolates are graphed as the mean ± standard deviation.
(TIF)

Table S1 Whole-genome transcriptional profiling results of *ryp* T-DNA mutants. Normalized expression ratios were analyzed with BAGEL using default parameters. Relative expression ratios and posterior probabilities for each pairwise comparison are listed for each gene. Phase-specific (YPS and FPS) genes were identified using the criteria of *p* value <0.01 and >2-fold change in gene expression levels in the WT_37 (wild-type yeast cells) versus WT_RT (wild-type filamentous cells) comparison. These genes are shown also as a heatmap in Figure 1A.
(TXT)

Table S2 ChIP-chip results. ChIP events identified in this study are listed according to their genomic locations. Each ChIP event was mapped to annotated genes with a criterion of an event preceding an open reading frame with 5′-3′ orientation. Target genes, associated ChIP event IDs, gene annotations, and Log2-based expression ratios (WT_37/WT_RT) are listed. This list was used to generate Figures 2 and S5.
(TXT)

Table S3 Whole-genome transcriptional profiling results of *ryp* knockdown strains. Normalized expression ratios were analyzed with BAGEL using default parameters. Relative expression ratios and posterior probabilities for each pairwise comparison are listed for each gene. Phase-specific (YPS and FPS) genes were identified using the criteria of *p* value <0.01 and >2-fold change in gene expression levels in the WT_37 (wild-type yeast cells) versus WT_RT (wild-type filamentous cells) comparison. These genes are shown also as a heatmap in Figure 4E.
(TXT)

Table S4 Motif position weight matrices. Position weight matrices of motifs shown in Figure S6A and S6B in Mochiview format. Each row corresponds to each nucleotide position and columns indicate weights for A, C, G, and T, respectively.
(TXT)

Table S5 Genomic coordinates of Motif A and Motif B hits. All intergenic sequences were searched for occurrences of Motif A and Motif B using MAST. Motif hits were filtered using <10% false positive rate as a cut-off (corresponding to *p* values of 2.08e-04 for Motif A and 9.18e-05 for Motif B).
(TXT)

Table S6 Plasmids used in this study.
(PDF)

Table S7 Primers used in this study.
(PDF)

## Abbreviations

ChIP: chromatin immunoprecipitation
FPS: filamentous-phase–specific
YPS: yeast-phase–specific
