# Supplementary figures and images for: A Temperature-Responsive Network Links Cell Shape and Virulence Traits in a Primary Fungal Pathogen
Source: PLoS Biol. 2013 Jul 23;11(7):e1001614. doi: 10.1371/journal.pbio.1001614 (PMC3720256; doi:10.1371/journal.pbio.1001614)

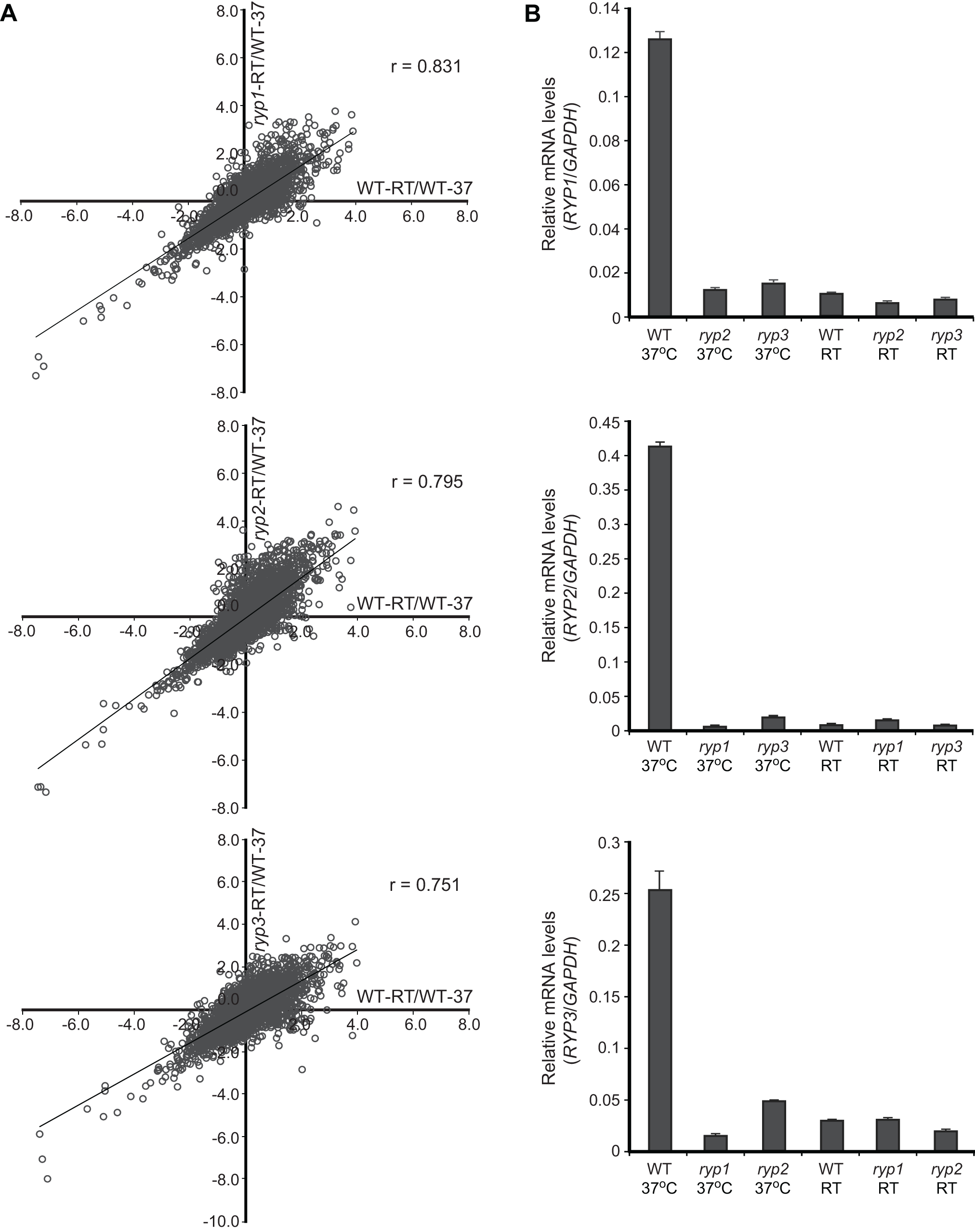

Supplement: Figure S1 — Ryp factors are required for each other's expression. (A) Correlation between transcriptional profiles of YPS and FPS genes in ryp mutants and wild-type cells grown at room temperature are presented as scatter plots with Pearson correlation coefficients (r, n = 9,289). (B) qRT-PCR was used to quantify relative levels of RYP1, RYP2, and RYP3 transcripts in ryp1, ryp2, and ryp3 mutants and wild-type cells grown at 37°C and room temperature (RT). GAPDH was used as a normalizer gene. Experiments were performed with at least two biological replicates for each strain. Triplicate measurements of representative replicates are graphed as the mean ± standard deviation. (TIF) [file pbio.1001614.s001.tif]

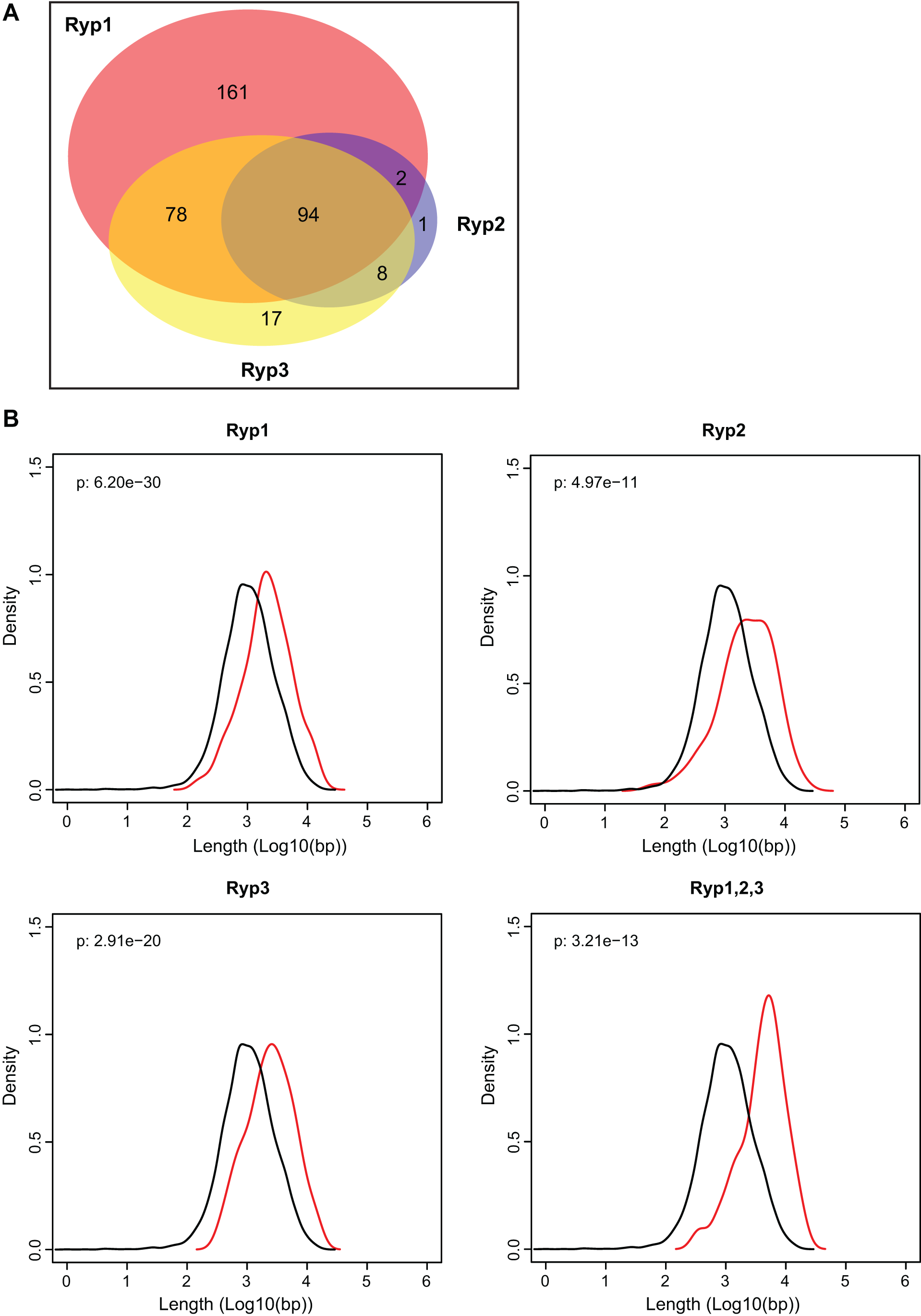

Supplement: Figure S2 — Ryp events occur at longer intergenic regions. (A) Numbers of Ryp1, Ryp2, and Ryp3 ChIP events are shown as a Venn diagram. (B) Intergenic length distributions for the whole genome (black line) and Ryp1, Ryp2, and Ryp3 individual and common ChIP events (red lines) are shown. The differences between length distributions were compared using the Wilcoxon test. (TIF) [file pbio.1001614.s002.tif]

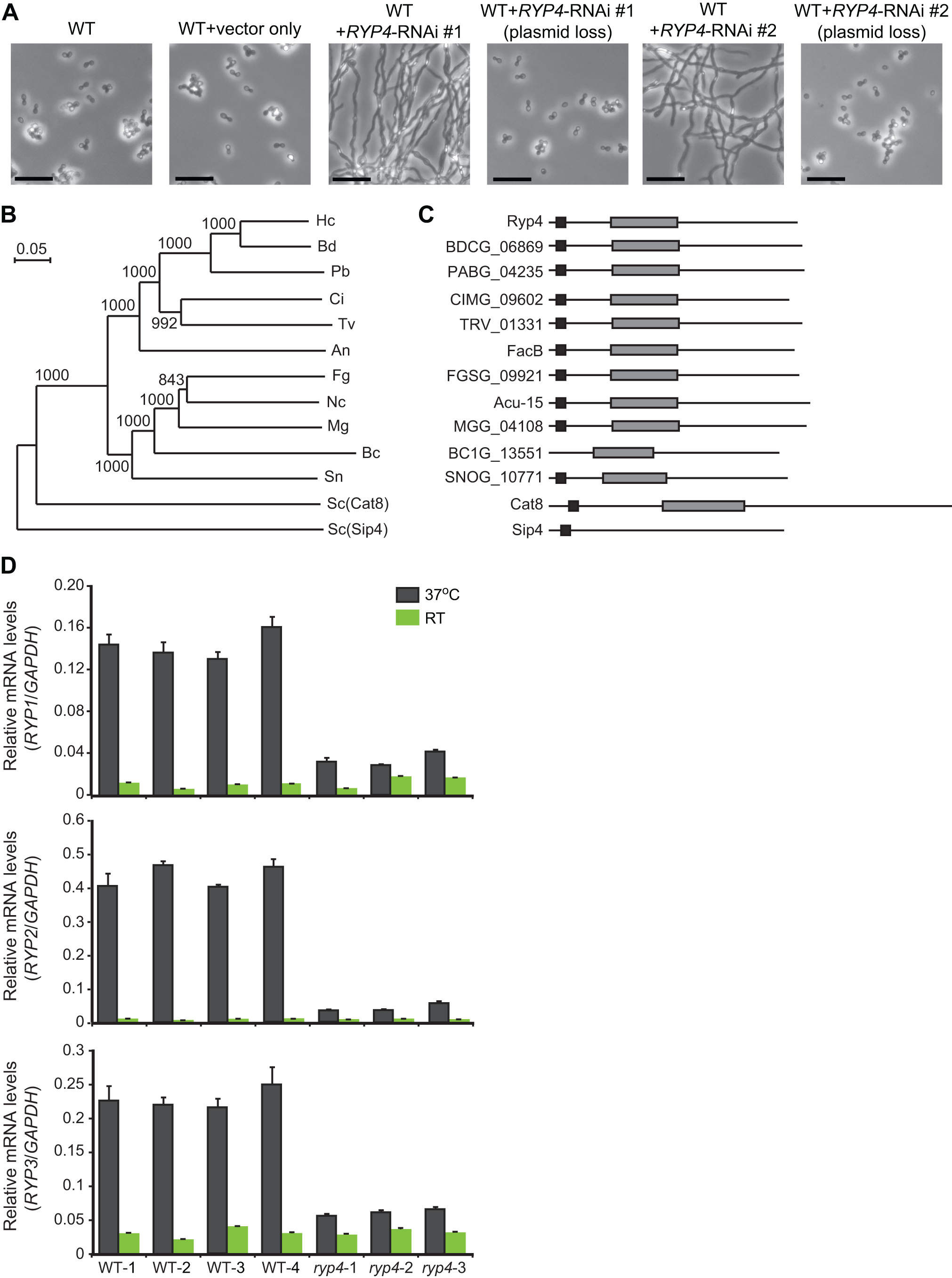

Supplement: Figure S3 — Ryp4 is required for yeast-phase growth and the expression of RYP genes. (A) Phase-contrast microscopy images of wild-type cells, or wild-type cells carrying either a vector control or RYP4 RNAi plasmid are shown. RNAi vectors were maintained episomally and strains were grown at 37°C. ryp4 knockdown strains were grown without selection to allow growth of clones that underwent spontaneous plasmid loss. Isolates of these strains that converted to the yeast form were analyzed to confirm that they had lost the RNAi marker. Black bar equals 20 µm. (B, C) Ryp4 is an ortholog of FacB. Initial sets of Ryp4 homologs were identified using HMMer 3.0 and the Pfam hidden Markov models (HMMs) for the fungal Zn(II)2Cys6 binuclear cluster domain (PF00172.13, shown in black boxes) and the fungal-specific transcription factor domain (PF04082.13, shown in grey boxes). Details of the databases used and searches are given in Materials and Methods. The full-length protein sequences of the Ryp4 orthologs, as well as Cat8 and Sip4, were aligned (C), and a bootstrapped (n = 1,000) neighbor-joining tree (B) was generated using CLUSTALW. (D) qRT-PCR was used to quantify relative levels of RYP1, RYP2, and RYP3 transcripts in ryp4 mutants and wild-type controls grown at 37°C and room temperature (RT). GAPDH was used as a normalizer gene. Experiments were performed with at least two biological replicates for each strain. Triplicate measurements of representative replicates are graphed as mean ± standard deviation. (TIF) [file pbio.1001614.s003.tif]

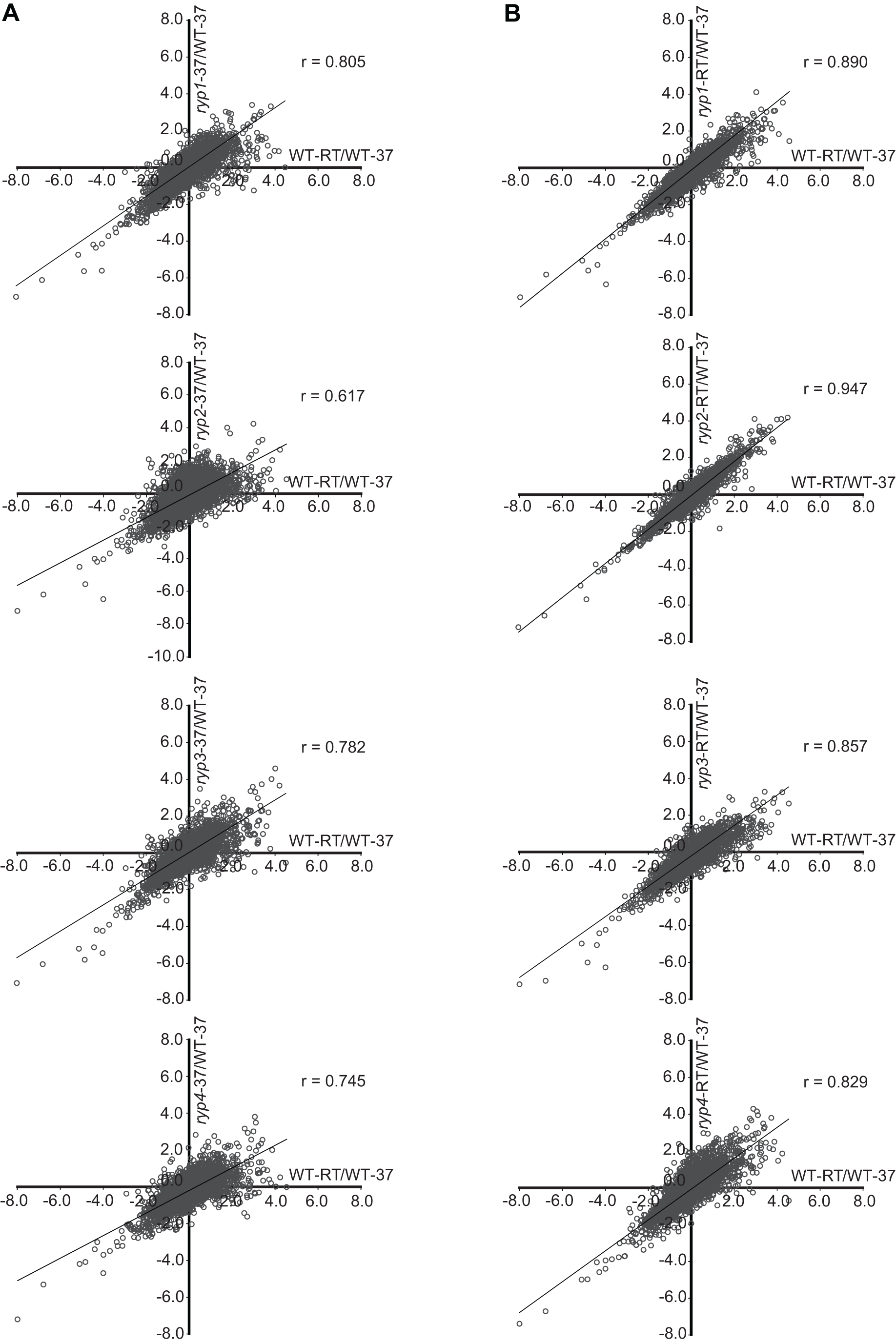

Supplement: Figure S4 — Ryp knockdown mutants display similar transcriptional profiles to that of wild-type filaments. Correlation between transcriptional profiles of YPS and FPS genes in ryp mutants grown at (A) 37°C and (B) room temperature and wild-type cells grown at room temperature are presented as scatter plots with Pearson correlation coefficients (r, n = 9,289). (TIF) [file pbio.1001614.s004.tif]

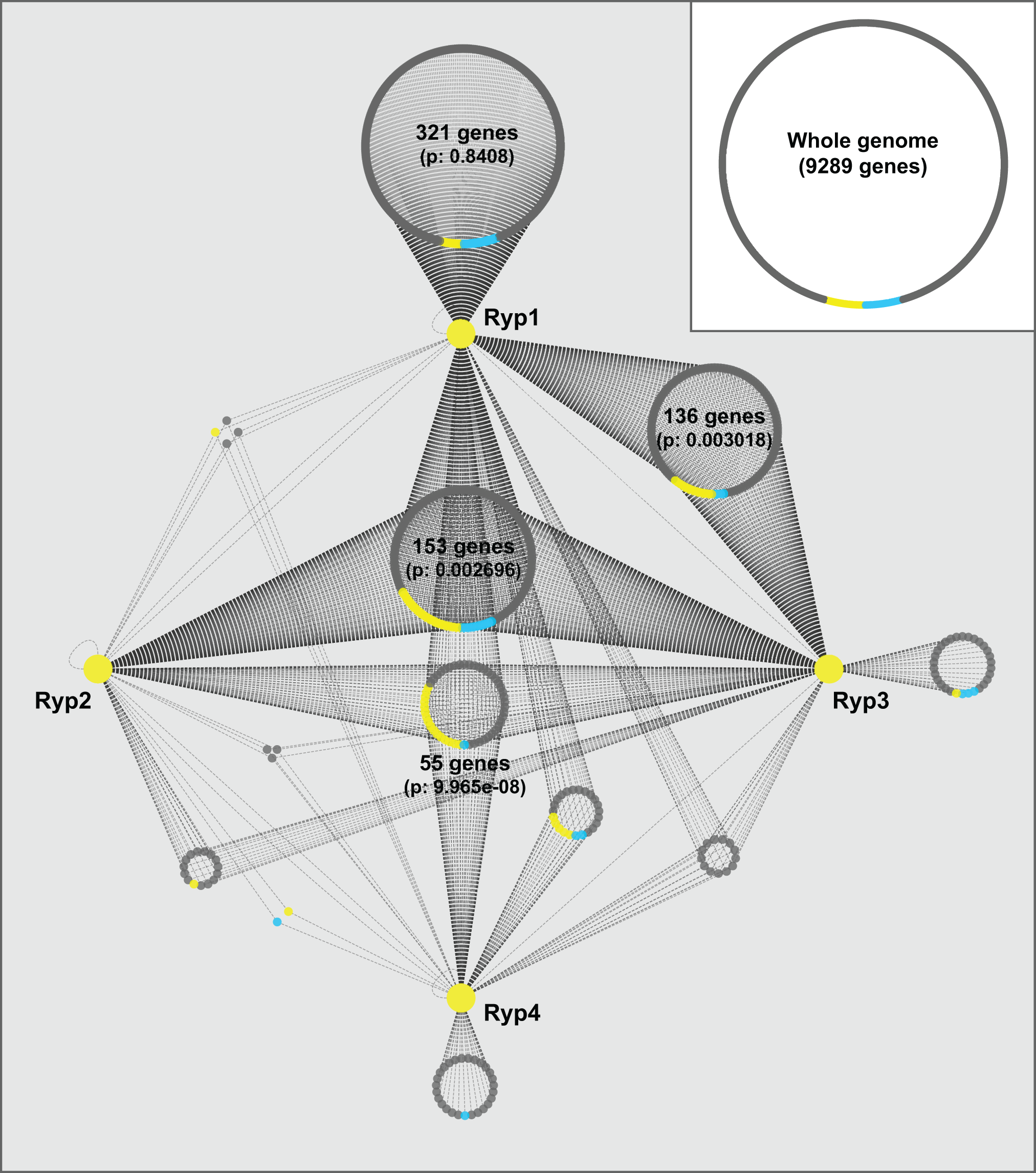

Supplement: Figure S5 — Ryp4 associates upstream of Ryp1, Ryp2, and Ryp3 targets. A network view of ChIP targets was generated using Cytoscape software. Each large open circle is composed of individual closed circles that represent individual genes. YPS genes are colored in yellow, and FPS genes are colored in blue. Microarrays used in this study represent 9,289 genes, which were also colored similarly as shown. The distribution of YPS and FPS genes in each type of ChIP event was compared to the whole genome using the Wilcoxon test. The p values obtained in this analysis are given. Additionally, Ryp1, Ryp3; Ryp1, Ryp2, Ryp3; and Ryp1, Ryp2, Ryp3, Ryp4 shared targets were enriched for YPS genes (p value <0.0001) as determined by hypergeometric tests performed in R. (TIF) [file pbio.1001614.s005.tif]

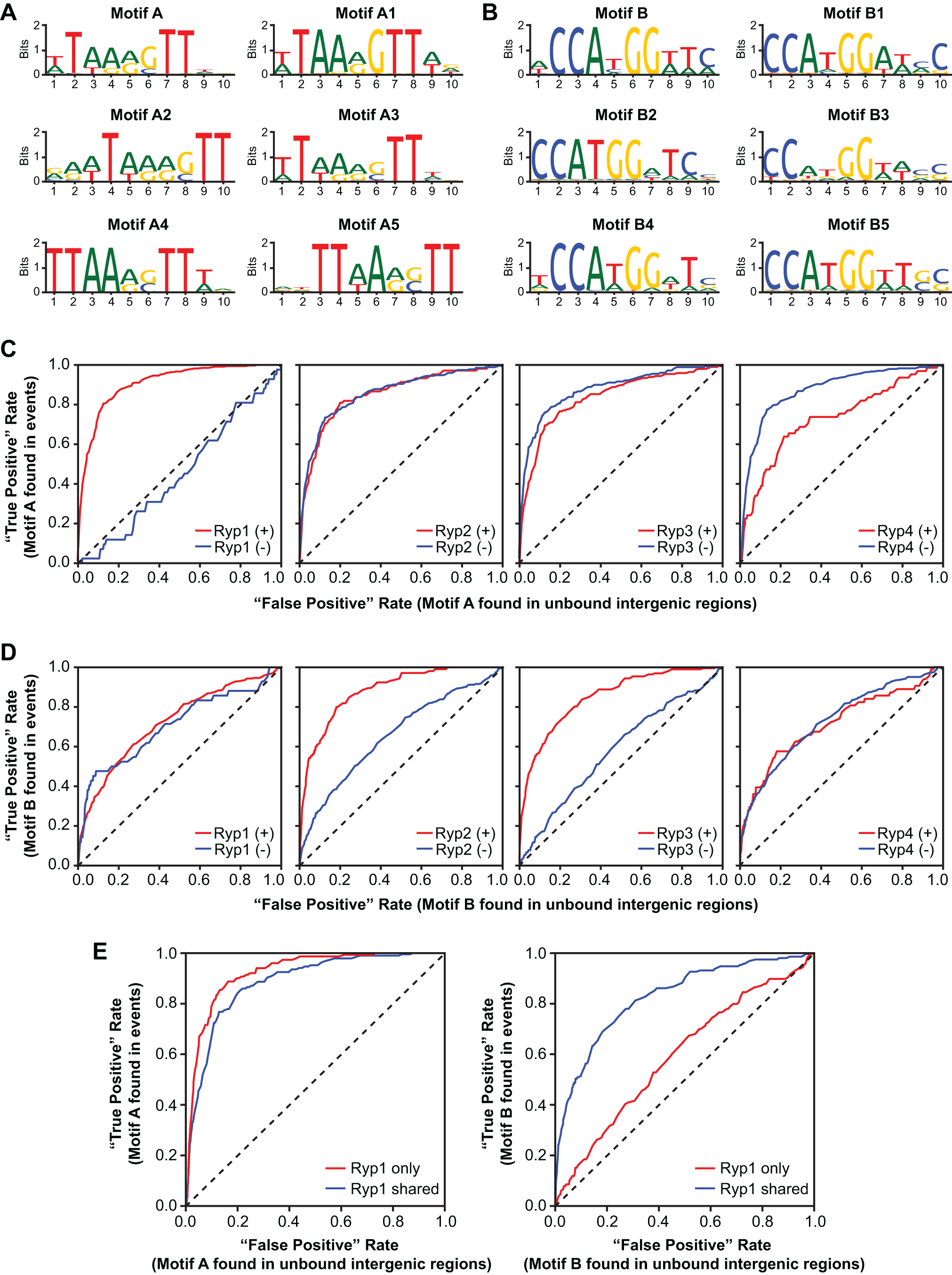

Supplement: Figure S6 — Ryp events are enriched for specific DNA sequences. Logos of (A) Motif A and (B) Motif B variants are shown. (C, D) Motif specificity of (C) Motif A and (D) Motif B were analyzed by ROC plots. True positive rate was defined as motifs found in ChIP events that contained or excluded a given Ryp event. False positive rate was defined as motifs found in unbound intergenic regions. (E) Similarly, motif specificity of Motif A and Motif B was analyzed in Ryp1-only or shared events by ROC plots. (TIF) [file pbio.1001614.s006.tif]

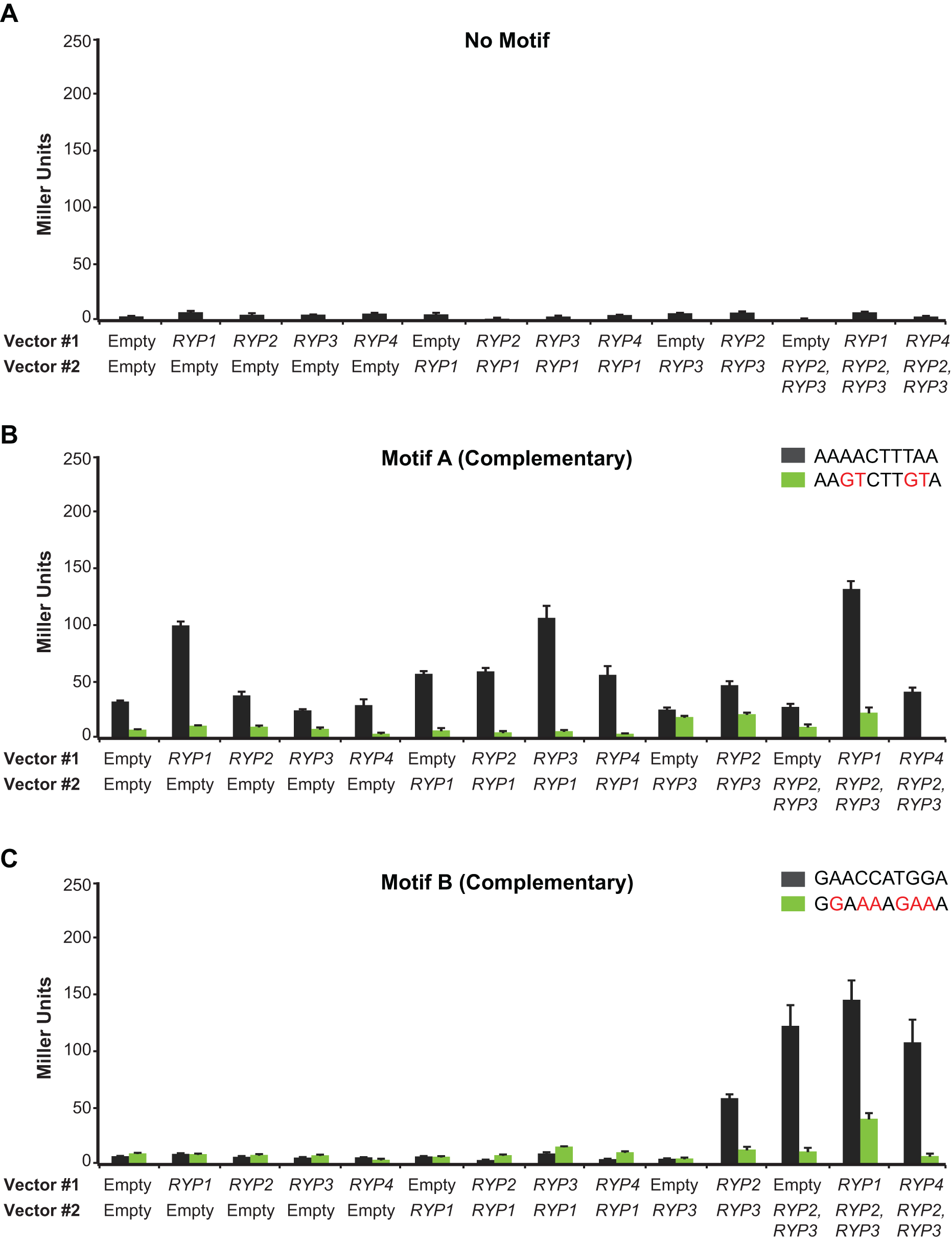

Supplement: Figure S7 — Motif A and Motif B are sufficient to drive gene expression in the presence of Ryp factors. RYP genes were cloned into two different vectors with two different markers. Each of these vectors were transformed into yeast strains with plasmids containing the UAS-less CYC1 promoter fused to the lacZ gene to generate “no motif” controls (A). Additionally, complementary sequences of (B) Motif A, (C) Motif B, and their mutated versions were cloned into a plasmid containing the UAS-less CYC1 promoter fused to the lacZ gene. Point mutations made in the motifs are shown in red. Each of the motif and Ryp plasmids were transformed into yeast strains as labeled. β-galactosidase activities were measured for three independent isolates of each strain. Quadruplicate measurements of representative isolates are graphed as the mean ± standard deviation. (TIF) [file pbio.1001614.s007.tif]
