# Supplementary material for: A Temperature-Responsive Network Links Cell Shape and Virulence Traits in a Primary Fungal Pathogen
Source: PLoS Biol. 2013 Jul 23;11(7):e1001614. doi: 10.1371/journal.pbio.1001614 (PMC3720256; doi:10.1371/journal.pbio.1001614)
Supplement: Table S6 — Plasmids used in this study. (PDF) [file pbio.1001614.s013.pdf]

Table S6. Plasmids used in this study

Plasmids used in RNAi experiments

Plasmid	Strain	Description	Reference/Source
pDONR/Zeo	BAS223	Donor vector for Gateway cloning system; Zeo^R	Invitrogen
pFANTAi4	BAS445	Destination vector with pH2AB divergent promoter driving Pa*URA5* and an RNAi sentinel system with GFP; Pa*URA5*; hyg^R; kan^R	(1)
pVN69	BAS611	Integrating RNAi control plasmid, lacks *ccdB* gene and Gateway recombination sites; Pa*URA5*; hyg^R; kan^R	(2)
pSB23	BAS538	Destination vector with pH2AB divergent promoter driving PaURA5 and an RNAi sentinel system with GFP; Pa*URA5*; kan^R	This Study
pCR186	BAS446	Episomal RNAi control plasmid, lacks *ccdB* gene and Gateway recombination sites; Pa*URA5*; kan^R	Dr. Chad A. Rappleye
pSB7	BAS522	pDONR/Zeo::*RYP4*-RNAi-E1; Zeo^R	This Study
pSB8	BAS523	pDONR/Zeo::*RYP4*-RNAi-E5; Zeo^R	This Study
pSB18	BAS533	pFANTAi4::*RYP4*-RNAi-E1; Pa*URA5*; hyg^R; kan^R	This Study
pSB19	BAS534	pFANTAi4::*RYP4*-RNAi-E5; Pa*URA5*; hyg^R; kan^R	This Study
pSB30	BAS545	pSB23::*RYP4*-RNAi-E1; Pa*URA5*; kan^R	This Study
pSB31	BAS546	pSB23::*RYP4*-RNAi-E5; Pa*URA5*; kan^R	This Study

Plasmids used in Yeast-two-Hybrid experiments

Plasmid	Strain	Description	Reference/Source
pEG202	BAS735	Empty bait plasmid for yeast-two-hybrid assays; *HIS3*; Amp^R	(3)
pJSC401	BAS736	Empty prey plasmid for yeast-two-hybrid assays; *TRP1*; Amp^R	(4)
pSB73	BAS791	pEG202::*RYP2*; *HIS3*; Amp^R	This Study
pSB74	BAS792	pEG202::*RYP3*; *HIS3*; Amp^R	This Study
pSB79	BAS797	pJSC401::*RYP2*; *TRP1*; Amp^R	This Study
pSB80	BAS798	pJSC401::*RYP3*; *TRP1*; Amp^R	This Study
pSB86	BAS826	pEG202::*RYP2* (1-624 bp); *HIS3*; Amp^R	This Study
pSB87	BAS827	pEG202::*RYP2* (595-1215bp); *HIS3*; Amp^R	This Study
pJW1	BAS1210	pJSC401::*RYP2* (1-624 bp); *TRP1*; Amp^R	This Study
pJW2	BAS1211	pJSC401::*RYP2* (595-1215 bp); *TRP1*; Amp^R	This Study

Plasmids used *in vivo* transcriptional activation assays

Plasmid	Strain	Description	Reference/Source
p413TEF	BAS872	P_{TEF}-(empty vector)-t_{CYC1}; *HIS3; CEN*; Amp^R	(5)
p414TEF	BAS873	P_{TEF}-(empty vector)-t_{CYC1}; *TRP1; CEN*; Amp^R	(5)
pSB93	BAS925	p413TEF::*RYP1*; *HIS3; CEN*; Amp^R	This Study
pSB94	BAS926	p414TEF::*RYP1*; *TRP1; CEN*; Amp^R	This Study
pSB95	BAS927	p414TEF::*RYP2*; *TRP1; CEN*; Amp^R	This Study
pSB97	BAS929	p413TEF::*RYP3*; *HIS3; CEN*; Amp^R	This Study
pSB98	BAS930	p414TEF::*RYP3*; *TRP1; CEN*; Amp^R	This Study
pSB99	BAS931	p414TEF::*RYP4*; *TRP1; CEN*; Amp^R	This Study
pSB115	BAS950	p413TEF::*RYP2-RYP3*; *HIS3; CEN*; Amp^R	This Study
pSB135	BAS1209	p414TEF::*RYP2* (1-624 bp); *TRP1; CEN*; Amp^R	This Study
P_{CYC1}-ΔUAS-*lacZ*	BAS878	Empty *lacZ* reporter plasmid (no motif); *URA3*; 2μ; Amp^R	(6)
p228	BAS933	P_{CYC1}-ΔUAS-*lacZ*::Motif A/Wor1 motif cloned in forward orientation (AAAAATTAAAGTTTTTTTAT); *URA3*; 2μ; Amp^R	(7)

p230	BAS934	P_{CYC1}-ΔUAS-*lacZ*::Motif A/Wor1 knock-out motif cloned in forward orientation (AAAAATACAAGACTTTTTAT); *URA3*; 2µ; Amp^R	(7)
pSB101	BAS935	P_{CYC1}-ΔUAS-*lacZ*::Motif A/Wor1 motif cloned in reverse orientation (ATAAAAAAACTTTAATTTTT); *URA3*; 2µ; Amp^R	This Study
pSB102	BAS936	P_{CYC1}-ΔUAS-*lacZ*::Motif A/Wor1 knock-out motif cloned in reverse orientation (ATAAAAAGTCTTGTATTTTT); *URA3*; 2µ; Amp^R	This Study
pSB107	BAS941	P_{CYC1}-ΔUAS-*lacZ*::Motif B cloned in forward orientation (ACTAGGTTCCATGGTTC); *URA3*; 2µ; Amp^R	This Study
pSB108	BAS942	P_{CYC1}-ΔUAS-*lacZ*::Motif B cloned in reverse orientation (GAACCATGGAACCTAGT); *URA3*; 2µ; Amp^R	This Study
pSB109	BAS943	P_{CYC1}-ΔUAS-*lacZ*::Motif B knock-out cloned in forward orientation (ACTAGGTTTTCTTTTCC); *URA3*; 2µ; Amp^R	This Study
pSB110	BAS944	P_{CYC1}-ΔUAS-*lacZ*::Motif B knock-out cloned in reverse orientation (GGAAAAGAAAACCTAGT); *URA3*; 2µ; Amp^R	This Study

Plasmids used to express and purify Ryp proteins for EMSAs

**Plasmid**	**Strain**	**Description**	**Reference/Source**
pSB122	BAS1164	pCR2.1-TOPO::*RYP1*-6XHis; Amp^R	This Study
pSB124	BAS1166	pCR2.1-TOPO::*RYP1*(1-801 bp)-6XHis; Amp^R	This Study
pSB128	BAS1170	pCR2.1-TOPO::*RYP2*-6XHis; Amp^R	This Study
pSB130	BAS1172	pCR2.1-TOPO::*RYP3*-6XHis; Amp^R	This Study

References:

1. Krajaejun T, Gauthier GM, Rappleye CA, Sullivan TD, Klein BS (2007) Development and application of a green fluorescent protein sentinel system for identification of RNA interference in Blastomyces dermatitidis illuminates the role of septin in morphogenesis and sporulation. Eukaryotic Cell 6(8):1299-1309.

2. Nguyen VQ, Sil A (2008) Temperature-induced switch to the pathogenic yeast form of *Histoplasma capsulatum* requires Ryp1, a conserved transcriptional regulator. Proc Natl Acad Sci USA 105(12): 4880-4885.

3. Golemis EA, Serebriiskii I, Finley RL, Kolonin MG, Gyuris J, Brent R (2001) Interaction Trap/Two-Hybrid System to Identify Interacting Proteins. Curr Protoc Mol Biol. 46:20.1.1–20.1.40

4. Stanley SA, Raghavan S, Hwang WW, Cox JS (2003) Acute infection and macrophage subversion by *Mycobacterium tuberculosis* require a specialized secretion system. Proc Natl Acad Sci USA 100(22):13001-13016.

5. Mumberg D, Müller R, Funk M (1995) Yeast vectors for the controlled expression of heterologous proteins in different genetic backgrounds. Gene 156(1):119-122.

6. Rupp S, Summers E, Lo HJ, Madhani H, Fink G. (1999) MAP kinase and cAMP filamentation signaling pathways converge on the unusually large promoter of the yeast *FLO11* gene. EMBO J 18(5):1257-1269.

7. Lohse MB, Zordan RE, Cain CW, Johnson AD (2010) Distinct class of DNA-binding domains is exemplified by a master regulator of phenotypic switching in *Candida albicans*. Proc Natl Acad Sci USA 107(32): 14105-14110.
