# Supplementary material for: A Temperature-Responsive Network Links Cell Shape and Virulence Traits in a Primary Fungal Pathogen
Source: PLoS Biol. 2013 Jul 23;11(7):e1001614. doi: 10.1371/journal.pbio.1001614 (PMC3720256; doi:10.1371/journal.pbio.1001614)
Supplement: Table S7 — Primers used in this study. (PDF) [file pbio.1001614.s014.pdf]

Table S7. Primers used in this study

Primers used to generate RNAi plasmids	
**Primer number**	**Sequence (5'-3')**
OAS2235 *RYP4*-E1 RNAi Fwd	GGGGACAAGTTTGTACAAAAAAGCAGGCTCCCATGAAGGTGATCAAGGT
OAS2236 *RYP4*-E1 RNAi Rev	GGGGACCACTTTGTACAAGAAAGCTGGGTGGGTGCATCTGTCGTAGGTT
OAS2237 *RYP4*-E5 RNAi Fwd	GGGGACAAGTTTGTACAAAAAAGCAGGCTACTGCCTCCCCTTCCATACT
OAS2238 *RYP4*-E5 RNAi Rev	GGGGACCACTTTGTACAAGAAAGCTGGGTGTCCGAAGAAAGCTCACCTG

Primers used in qPCR experiments	
**Primer number**	**Sequence (5'-3')**
OAS1057 *RYP1*-qPCR-Fwd[1]	ACCCTTGCAGCTTACAACCT
OAS1058 *RYP1*-qPCR-Rev[1]	TCCGTCCATCGCTTAATACC
OAS1942 *RYP2*-qPCR-Fwd[1]	CGGCTCGAGAGATGAAGTCGTT
OAS1943 *RYP2*-qPCR-Rev[1]	AAGTGTACGGGCTTCCTTCCG
OAS1944 *RYP3*-qPCR-Fwd[1]	CCAAAGGCCAAGATGGAGAAGG
OAS1945 *RYP3*-qPCR-Rev[1]	GGAAATGAGAGGAAGGGGGAAAGA
OAS3320 *RYP4*-qPCR-Fwd	GAACTTGATGAGTGGCAGAGG
OAS3321 *RYP4*-qPCR-Rev	ACTAGGGCCAGTATGGAAGG
OAS1452 *GAPDH*-qPCR-Fwd	AGACCCACTATGCTGCCTACA
OAS1453 *GAPDH*-qPCR-Rev	GGGTCGTATGTTTTCTCGT

Primers used to generate Ryp expression plasmids	
**Primer number**	**Sequence (5'-3')**
OAS3411 *RYP1*-Fwd-EcoRI	GGAATTCATGGGCAACGGCACAGCTG
OAS3549 *RYP1*-Rev-XhoI	CCGCTCGAGTCAACCTGTTGCAGCCGTATTCC
OAS3555 *RYP2*-Fwd-BamHI	CGGGATCCGTATGAGCGCGCCAACATTTGC
OAS3750 *RYP2*-Rev-SalI	GGAGTCGACTCATCCCCATGCCGGCG
OAS3415 *RYP3*-Fwd-EcoRI	GGAATTCATGTACACGCTCAAACAAGACCGC
OAS3551 *RYP3*-Rev-XhoI	CCGCTCGAGTCACCCTTCATTCTCCCAATCCTC
OAS3751 *RYP4*-Fwd-SpeI	GACTAGTATGCCCGGCATACTACCCATG
OAS3752 *RYP4*-Rev-SalI	GGAGTCGACTCACGCAGGTTGTCGTAAATCCC
OAS3565 pEG202-Fwd	ATTGAAGGGCTGGCGGTTGGGGTTATTCGCAACGGCGACTGGCTGGAATTC
OAS3566 pEG202-Rev	TAAATCATAAGAAATTCGCCCGGAATTAGCTTGGCTGCAGGTCGACTCGAG
OAS3559 *RYP2*-pEG202-Fwd	GGCGACTGGCTGGAATTCATGAGCGCGCCAACATTTGC
OAS3560 *RYP2*-pEG202-Rev	GCTGCAGGTCGACTCGAGTCATCCCCATGCCGGCG
OAS3595 *RYP2*-C-pEG202-Fwd	GGCGACTGGCTGGAATTCACACTTCTAAAACGCCCATTGAATCG
OAS3596 *RYP2*-N-pEG202-Rev	GCTGCAGGTCGACTCGAGTCACGCTCGATTCAATGGGCGTT
OAS3573 pJSC401-Fwd	GGAGATGCCTCCTACCCTTATGATGTGCCAGATTATGCCTCTCCCGAATTC
OAS3574 pJSC401-Rev	TGATTGGAGACTTGACCAAACCTCTGGCGAAGAAGTCCAAAGCTTCTCGAG
OAS3567 *RYP2*-pJSC401-Fwd	TATGCCTCTCCCGAATTCATGAGCGCGCCAACATTTGC
OAS3568 *RYP2*-pJSC401-Rev	AGTCCAAAGCTTCTCGAGTCATCCCCATGCCGGCG
OAS3783 TEFprom-SacI-Fwd	CTGGAGCTCATAGCTTCAAAATGTTTCTAC
OAS3784 CYC1term-SacI-Rev	CTGGAGCTCGGCCGCAAATTAAAGCCTTCG

OAS4031 *RYP2*-C-pJSC401-Fwd	TATGCCTCTCCCGAATTCACACTTCTAAAACGCCCATTGAATCG
OAS4032 *RYP2*-N-pJSC401-Rev	AGTCCAAAGCTTCTCGAGTCACGCTCGATTCAATGGGCGTT
OAS4205 *RYP2*-N-Rev-SalI	GGAGTCGACTCATTACGCTCGATTCAATGGGCGTT

Primers used to generate motif plasmids

Primer number	Sequence (5'-3')
OAS3893/MBL1039[2]	TCGAGAAAAATTAAAGTTTTTTTATC
OAS3894/MBL1040[2]	TCGAGATAAAAAAACTTTAATTTTTC
OAS3895/MBL1045[2]	TCGAGAAAAATACAAGACTTTTTATC
OAS3896/MBL1046[2]	TCGAGATAAAAAGTCTTGTATTTTTC
OAS3901 MotifB-Fwd	TCGAGACTAGGTTCCATGGTTCC
OAS3902 MotifB-Rev	TCGAGGAACCATGGAACCTAGTC
OAS3903 MotifB-KO-Fwd	TCGAGACTAGGTTTTCTTTTCCC
OAS3904 MotifB-KO-Rev	TCGAGGGAAAAGAAAACCTAGTC

Primers used to generate plasmids expressing His-tagged Ryp proteins

Primer number	Sequence (5'-3')
OAS4102 6XHis-Fwd	GATCTTAAGGCTAGAGTACTAATACGACTC
OAS4103 6XHis-Rev	AAAAAACCCCTCAAGACCCG
OAS4106 Cterm-6XHis-Fwd	GATCTTAAGGCTAGAGTACTAATACGACTCACTATAGGGAATACAAGCTACTTGT TCTTTTTGCACCACC
OAS4107 Cterm-6XHis-Rev	AAAAAACCCCTCAAGACCCGTTTAGAGGCCCCAAGGGGTTTTTTTTTTTTTTTTT TTTTTTTTTTTTTCATTAATGATGATGATGATGATGTCCCTGAAAATACAGGTTTTC
OAS4110 *RYP1*-N-6XHis-C-Fwd	GCTACTTGTTCTTTTTGCACCACCATGGGCAACGGCACAGCT
OAS4111 *RYP1*-N-6XHis-C-Rev	GATGTCCCTGAAAATACAGGTTTTCATATGACGGACCGGAACTCGG
OAS4114 *RYP1*-6XHis-C-Fwd	GCTACTTGTTCTTTTTGCACCACCATGGGCAACGGCACAGCT
OAS4115 *RYP1*-6XHis-C-Rev	GATGTCCCTGAAAATACAGGTTTTCACCTGTTGCAGCCGTATTCCA
OAS4118 *RYP2*-6XHis-C-Fwd	GCTACTTGTTCTTTTTGCACCACCATGAGCGCGCCAACATTTG
OAS4119 *RYP2*-6XHis-C-Rev	GATGTCCCTGAAAATACAGGTTTTCTCCCCATGCCGGCG
OAS4122 *RYP3*-6XHis-C-Fwd	GCTACTTGTTCTTTTTGCACCACCATGTACACGCTCAAACAAGACCG
OAS4123 *RYP3*-6XHis-C-Rev	GATGTCCCTGAAAATACAGGTTTTCCCCTTCATTCTCCCAATCCTCC

Oligonucleotides used to generate EMSA probes

Oligo name	Sequence (5'-3')
*CBP1*p-MotifA-Fwd	TAATGGTTCCAGATTGAGTTTCGTTTAAAGTTCACAACTCAATTCGGCAGCTCCGCTGCA
*CBP1*p-MotifA-Rev	TGCAGCGGAGCTGCCGAATTGAGTTGTGAACTTTAAACGAAACTCAATCTGGAACCATTA
*CBP1*p-MotifB-Fwd	ATCAGGCGTCCGATCAGGTGATTGCTTTTAACCTTGGGATCCAGAGATCTCTCTATCTTT
*CBP1*p-MotifB-Rev	AAAGATAGAGAGATCTCTGGATCCCAAGGTTAAAAGCAATCACCTGATCGGACGCCTGAT

[1]: Webster RH, Sil A (2008) Conserved factors Ryp2 and Ryp3 control cell morphology and infectious spore formation in the fungal pathogen *Histoplasma capsulatum*. Proc Natl Acad Sci USA 105(38): 14573-14578.
[2]: Lohse MB, Zordan RE, Cain CW, Johnson AD (2010) Distinct class of DNA-binding domains is exemplified by a master regulator of phenotypic switching in *Candida albicans*. Proc Natl Acad Sci USA 107(32): 14105-14110.
